# Structural basis for specific flagellin recognition by the NLR protein NAIP5

**DOI:** 10.1038/cr.2017.148

**Published:** 2017-11-28

**Authors:** Xinru Yang, Fan Yang, Weiguang Wang, Guangzhong Lin, Zehan Hu, Zhifu Han, Yijun Qi, Liman Zhang, Jiawei Wang, Sen-Fang Sui, Jijie Chai

**Affiliations:** 1 Innovation Center for Structural Biology, Tsinghua-Peking Joint Center for Life Sciences, School of Life Sciences, Tsinghua University, Beijing 100084, China; 2 Center for Plant Biology, School of Life Sciences, Tsinghua University, Beijing 100084, China; 3 State Key Laboratory of Membrane Biology, Innovation Center for Structural Biology, School of Life Sciences, Tsinghua University, Beijing 100084, China; 4 Department of Biological Chemistry and Molecular Pharmacology, Harvard Medical School, and Program in Cellular and Molecular Medicine, Boston Children's Hospital, Boston, MA 02115, USA; 5 Max-Planck Institute for Plant Breeding Research, 50829 Cologne, Germany; 6 Institute of Biochemistry, University of Cologne, Zuelpicher Str. 47, 50674 Koeln, Germany

**Keywords:** flagellin, NAIP5, NLRC4, cryo-EM

## Abstract

The nucleotide-binding domain- and leucine-rich repeat (LRR)-containing proteins (NLRs) function as intracellular immune receptors to detect the presence of pathogen- or host-derived signals. The mechanisms of how NLRs sense their ligands remain elusive. Here we report the structure of a bacterial flagellin derivative in complex with the NLR proteins NAIP5 and NLRC4 determined by cryo-electron microscopy at 4.28 Å resolution. The structure revealed that the flagellin derivative forms two parallel helices interacting with multiple domains including BIR1 and LRR of NAIP5. Binding to NAIP5 results in a nearly complete burial of the flagellin derivative, thus stabilizing the active conformation of NAIP5. The extreme C-terminal side of the flagellin is anchored to a sterically constrained binding pocket of NAIP5, which likely acts as a structural determinant for discrimination of different bacterial flagellins by NAIP5, a notion further supported by biochemical data. Taken together, our results shed light on the molecular mechanisms underlying NLR ligand perception.

## Introduction

The nucleotide-binding domain (NBD)- and leucine-rich repeat (LRR)-containing (NLR) proteins are a family of intracellular receptors that play an important role in regulation of innate immune response^1,2,3,4^. NLR proteins share a conserved tripartite domain structure with an N-terminal protein-protein interaction domain, a central nucleotide-binding and oligomerization domain (NOD) and a variable number of C-terminal LRR^1^. Similar domain structure is also present in plant NLR-type receptors^5^. In animals, several NLRs have been shown to function as pattern recognition receptors (PRRs), detecting pathogen-associated molecules patterns (PAMPs) or host-derived danger signals in the cytosol and consequently initiating innate immune response^3,6^. Following ligand perception, these immune NLRs oligomerize to form multiprotein complexes termed inflammasomes for activation of caspase-1^7,8^. Once activated, caspase-1 promotes proteolytic cleavage and secretion of IL-1β and IL-18. The activated caspase-1 can also cleave the substrate gasdermin D to induce pyroptosis, an inflammatory form of cell death^9,10,11^. Despite the important roles of NLRs in detecting the presence of pathogens in both animals and plants^5^, the mechanisms of how NLRs sense pathogen-derived ligands still remain poorly understood.

Neuronal apoptosis inhibitory protein (NAIP)-NLR containing a caspase activating and recruitment domain (CARD) 4 (NLRC4) complexes are ones of the most fully characterized NLR-containing inflammasomes^8,12,13,14,15,16,17^. In addition to the conserved NOD and LRR domains, NAIPs also contain three tandem baculovirus inhibitor of apoptosis protein repeats (BIRs) at their N-terminal sides^18^. The NAIP-NLRC4 inflammasomes are activated in macrophages infected by bacterial pathogens carrying flagellin or components of type III secretion system (T3SS)^19,20,21,22,23,24,25,26,27,28,29,30^. In mice, the specificity of the inflammasomes is conferred by NAIPs, with NAIP5/6 and NAIP2 recognizing bacterial flagellin and the T3SS component PrgJ, respectively^12,13^. NAIP1from mice and its human ortholog of hNAIP serve as receptors for T3SS needle proteins^26,27^. Like NAIP2/5, NAIP1 and hNAIP also form ligand-induced NAIP-NLRC4 inflammasomes for activation of caspase-1. A recent study suggested that the NBD-associated central domains other than LRR of NAIPs are important for their specific recognition of the two bacterial PAMPs^31^. The BIRs of NAIP5 have also been shown to be critical for flagellin-induced activation of NAIP5-NLRC4 inflammasome^12,13^, although how they contribute to the activity remains unclear. The highly conserved C-terminal 35 residues of flagellins are both necessary and sufficient to induce NLRC4-mediated immune response^21^. Interestingly, however, their activity of inducing NAIP5-NLRC4 inflammasomes varies dramatically^13^. Ligand recognition results in NAIP interaction with NLRC4 and consequent NLRC4 activation^12,13^. Once activated, NLRC4 self-propagates its active conformation, forming a wheel-like^14,15^ or spiral^16^ structure of NAIP-NLRC4 inflammasomes containing one NAIP and multiple NLRC4 molecules.

In the current study, we solved the cryo-EM structure of an active flagellin derivative^32^ in complex with the NLR proteins NAIP5 and NLRC4 at 4.28 (Å) angstrom. The structure revealed that the flagellin forms two helices and interacts with multiple domains of NAIP5, including N-terminal domain (NTD), BIR1, helical domain 1(HD1), insertion domain (ID) and LRR of NAIP5, which functions to stabilize the active conformation of NAIP5. The C-terminal helix of the flagellin dominates the interaction with NAIP5, with its extreme C-terminal side binding to a narrow pocket formed by BIR1 and HD1 of NAIP5. Supported by biochemical data, this pocket is important for the differential recognition of flagellins by NAIP5. Taken together, our data provide the structural mechanism of how an NLR protein recognizes its ligand.

## Results

### Cryo-EM structure of a flagellin-induced heterodimeric NAIP5-NLRC4 complex

**Figure 1:**
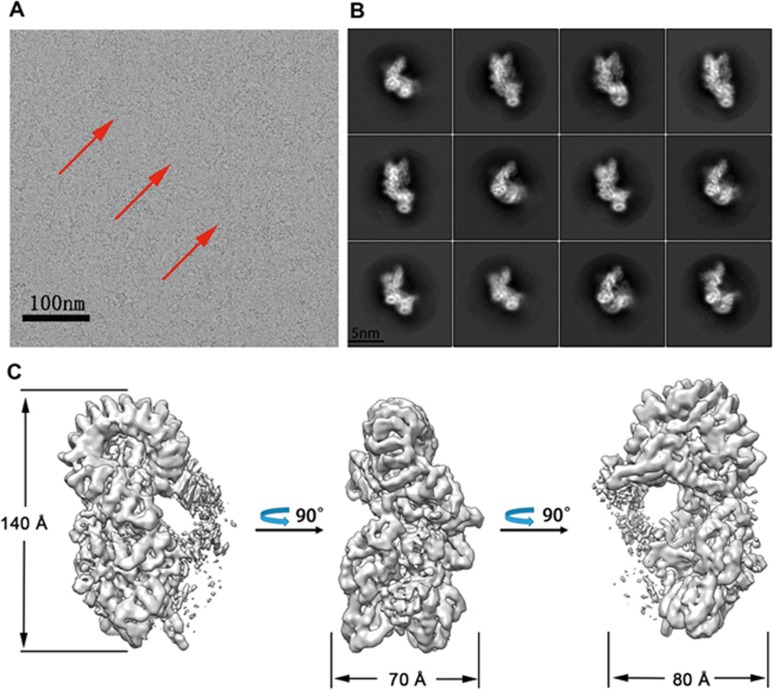
Cryo-EM analysis of FliC_D0_L_-NAIP5-NLRC4^M^ complex. **(A)** A representative electron micrograph of the FliC_D0_L_-NAIP5-NLRC4^M^ complex with low pass filtered to 5 Å. A few typical particles of the complex protein are marked by red arrows. NLRC4^M^, NLRC4^R288A-L435D-1 008-1 012DDYD-AAAA^. FliC_D0_L_, An *S. typhimurium* flagellin mutant with its N- and C-terminal sides connected by the linker “SGSGSG”. **(B)** Typical reference-free 2D class averages from the single-particle images of the FliC_D0_L_-NAIP5-NLRC4^M^ complex. **(C)** Three different views of the final EM density map of the FliC_D0_L_-NAIP5-NLRC4^M^ complex.

To determine how NAIP5 recognizes flagellin, we first purified an *Salmonella typhimurium* (*S. typhimurium*) flagellin derivative with its N- and C-terminal regions fused together (called FliC_D0_L_)^32^ in complex with wild-type NAIP5 and an NLRC4 carrying the mutations R288A-L435D-1 008-1 012DDYD-AAAA (called NLRC4^M^) from insect cells (Supplementary information, Figures S1 and S6A) as previously described^14^. The protein purified was then used for structural analysis with cryo-EM (Figure 1; Supplementary information, Figure S2). After 3D classification, a subset of 1 663 317 particles was used for image reconstruction, generating a map with a global resolution of 4.28 Å (Supplementary information, Figures S2, S3 and Table S1), as determined with a gold standard Fourier shell correlation (FSC) (Supplementary information, Figure S3B). The resolution is anisotropic (Supplementary information, Figure S3C) with BIR1, NBD, HD2, WHD, the unannotated domain^24^ (residues 921-980, call “ID” hereafter) and FliC_D0_L_ at resolution of ∼3.8-4.5 Å as supported by the visibility of larger side chains (Supplementary information, Figures S4 and S5). In contrast, LRR, BIR2 and the region N-terminal to BIR1 (residues 1-60, called “NTD” hereafter) have a lower resolution, in the range of ∼5-8 Å. Compared to NAIP5, NLRC4^M^ is less well defined in the 3D reconstruction (Supplementary information, Figure S2). Structural comparison between NAIP5-NLRC4^M^ and a lateral NLRC4 dimer from NLRC4 inflammasome^14,15,16^ showed that a conserved set of structural elements (from NBD and WHD) of NAIP5 and NLRC4 was involved in interaction with NLRC4 (Supplementary information, Figure S6). We therefore limit our discussion to NAIP5 interaction with FliC_D0_L_.

### Structure of the FliC_D0_L_-bound NAIP5 and its comparison with that of active NLRC4

**Figure 2:**
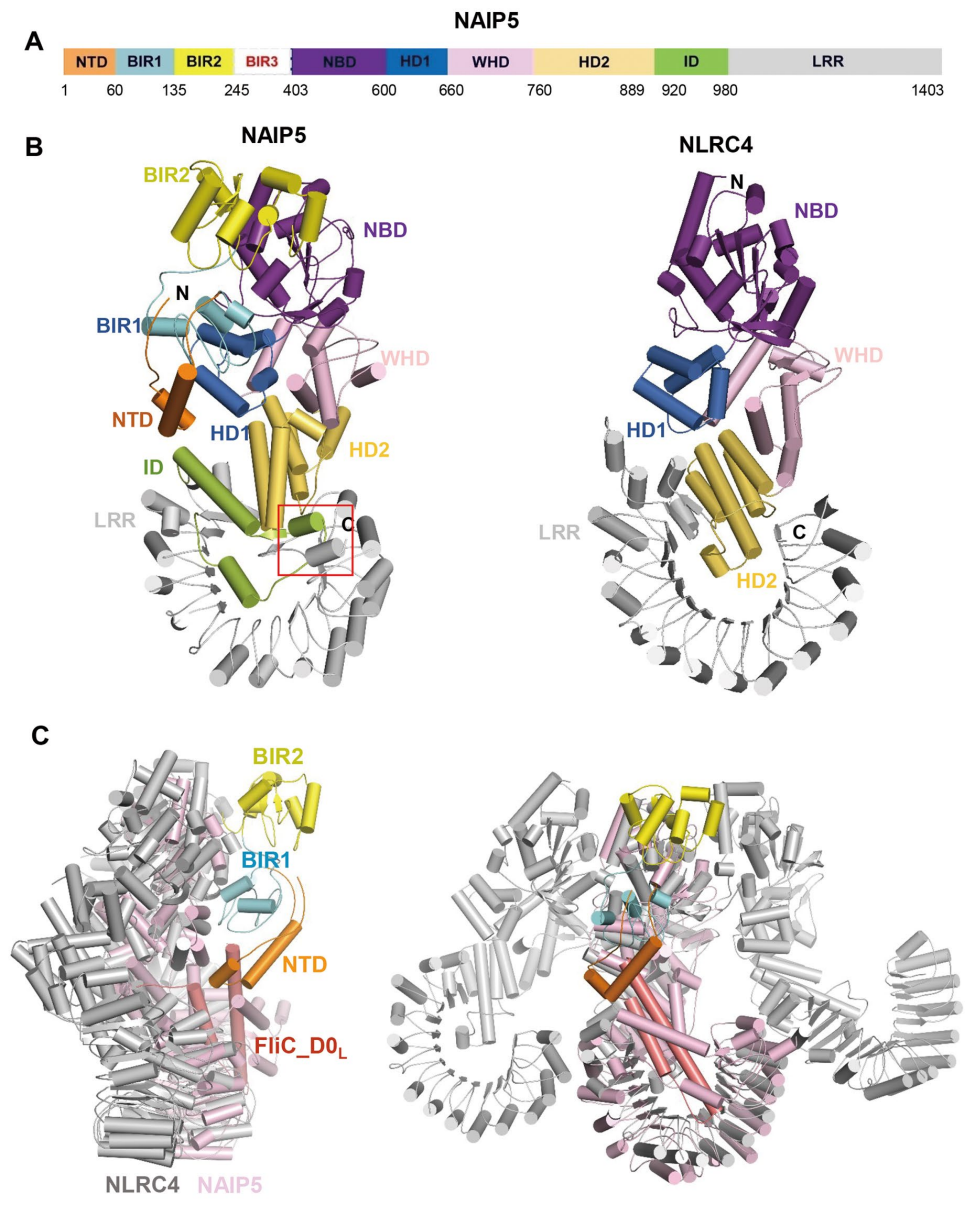
Atomic model of the FliC_D0_L_-bound NAIP5. **(A)** Schematic representation of the domain structures of NAIP5. Color codes for domains are indicated. Numbers indicate the domain boundaries. **(B)** Cartoon representations of active NAIP5 (left panel) and active NLRC4 (right panel). The aligned NAIP5 and NLRC4 are shown in the same orientation. Red frame indicates the location where C-terminal of NAIP5 packs with middle helix of ID. **(C)** Structural superposition of a lateral NLRC4 trimer from NLRC4 inflammasome with NAIP5 shown in two different orientations. NAIP5 was aligned with the middle NLRC4 protomer. For clarity, NTD, BIR1 and BIR2 are shown in the same colors as in **B** and labeled and all the other domains of NAIP5 are shown in pink. ID, insertion domain; NTD, N terminal domain.

The NBD, HD1, WHD, HD2 and LRR domains of NAIP5 (Figure 2A) are similarly positioned to those of an active NLRC4 (Figure 2B), indicating the structure of NAIP5 represents an active state. Located between NBD and HD1, clear electron density that is not from NAIP5 likely defines an ATP molecule (Supplementary information, Figure S4A and S4B). The ATP is coordinated by residues exclusively from NBD and HD1 of NAIP5 (Supplementary information, Figure S4C). BIR1 and BIR2 are juxtaposed at one side of NAIP5 (Figure 2B, left panel), with the former making contacts with HD1 and the latter with NBD. Compared to BIR1 and BIR2, BIR3 of NAIP5 is much less well defined in the 3D reconstruction and only fuzzy density is observed within the surface groove created by BIR1, BIR2, NBD1 and HD1 (Supplementary information, Figure S7A and S7B). The BIR3 domain is located at the opposite side of the NAIP5 surface interacting with an active NLRC4^14,15,16^ and could play a role in contacting the last NLRC4 molecule for closure of the ring-like NAIP-NLRC4 inflammasome as suggested by structural comparison (Supplementary information, Figure S7C). The structures of BIR1 and BIR2 of NAIP5 resemble that of BIR3 from XIAP^33^, but the peptide binding sites in these two BIR domains are completely blocked (Supplementary information, Figure S8).

Although structural domains are similarly positioned in the active NLRC4 and NAIP5, striking structure differences between them exist. In addition to NTD and the BIR domains, NAIP5 also contains an ID and extra 19 residues at its C-terminal side. The ID is mainly composed of three helices, with the middle one packing against the structural elements formed by the additional 19 residues of NAIP5 (Figure 2B, left panel). The middle helix and its surrounding regions of ID also contact one lateral side of the extreme C-terminal region of LRR, resulting in closure of the horse-shoe-like structure of the LRR domain. Another striking difference between NAIP5 and NLRC4 occurs in the regions around the conserved four-helical bundle of HD2 (Figure 2B). In the active NLRC4, a short helix is extended to establish interaction with the inner surface of its LRR (Figure 2B, right panel). By comparison, two helices pack against the conserved four-helical bundle of HD2 at a different side and interact with HD1 and WHD of NAIP5 (Figure 2B, left panel). The simultaneous interactions of these two NAIP5 helices with HD1 and WHD are expected to stabilize the active conformation of NAIP5, because striking structural reorganization between these two domains has been demonstrated for NLRC4 during activation^14,15,16^.

Structural alignment of NAIP5 with a lateral trimer from the NLRC4 inflammasome showed that the NAIP5-bound FliC_D0_L_ unlikely interacted with NLRC4 directly (Figure 2C), suggesting that the flagellin derivative allosterically activates NLRC4 to assemble NAIP5-NLRC4 inflammasome. This structural comparison also revealed that BIR1 and BIR2 are located far from the NAIP5-NLRC4 dimeric interface, indicating that these two domains are less likely to make a direct contribution to NAIP5-NLRC4 oligomerization.

### Overall structure of the FliC_D0_L_-NAIP5 complex

**Figure 3:**
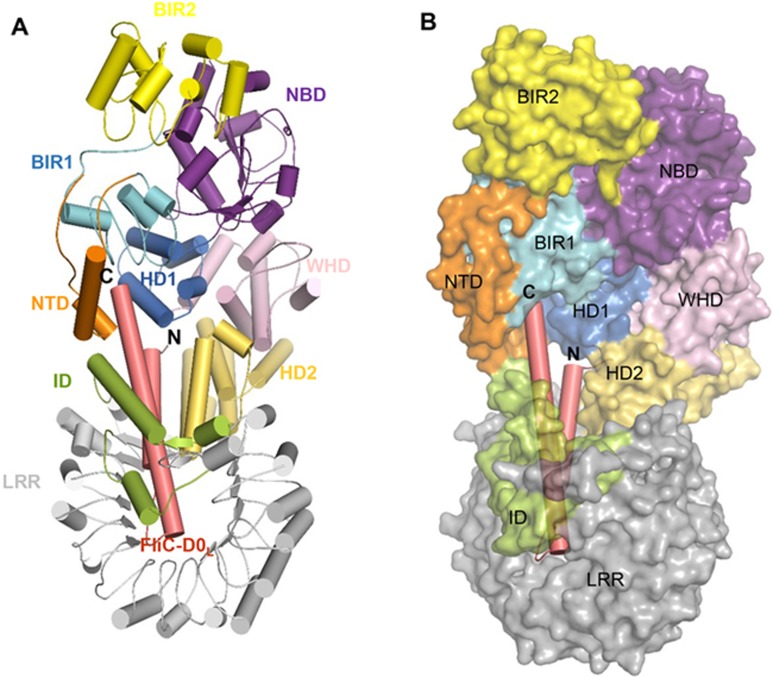
Overall structure of FliC_D0_L_-NAIP5 complex. **(A)** Cartoon representation of the FliC_D0_L_-NAIP5 complex. “N” and “C” represent N- and C-terminus, respectively. **(B)** Transparent surface and cartoon representations of NAIP5 and FliC_D0_L_, respectively. NAIP5 is shown with the same orientation as that shown on **A**.

The N- and C-terminal sides of FliC_D0_L_ (termed FliC-N and FliC-C, respectively) form two parallel α helices with few interactions formed between them (Figure 3A), as observed in the structure of full-length flagellin^34^. Both of the two helices interact with NAIP5, resulting in a nearly complete burial of FliC_D0_L_ (Figure 3B). FliC-C forms extensive interactions with NAIP5 via packing against NTD, BIR1, HD1, HD2, ID and LRR of NAIP5 (Figure 3B), supporting the observation that the C-terminal 35 resides of flagellin are necessary and sufficient for NAIP5 activation^21^. Participation of the non-conserved NTD, BIR1 and ID domains in interaction with FliC-C explains specific recognition of flagellin by NAIP5 but not by NLRC4. Compared to FliC-C, FliC-N forms much less dense contacts with NAIP5 (Figure 3B). The helical portion of FliC-N is sandwiched between HD2 and the inner surface of LRR of NAIP5 ((Figure 3), although identities of the residues from LRR cannot be unambiguously determined in the density map. In addition, the N-terminal side of FliC-N binds to a surface groove formed by HD1 and HD2 (Figure 3B), which likely also contributes to stabilization of the active conformation of NAIP5. Interestingly, NBD and WHD that are important for oligomerization of the NAIP5-NLRC4 inflammasome^14,15,16^ are not involved in NAIP5 recognition of FliC_D0_L_.

### Recognition mechanism of FliC_D0_L_ by NAIP5

**Figure 4:**
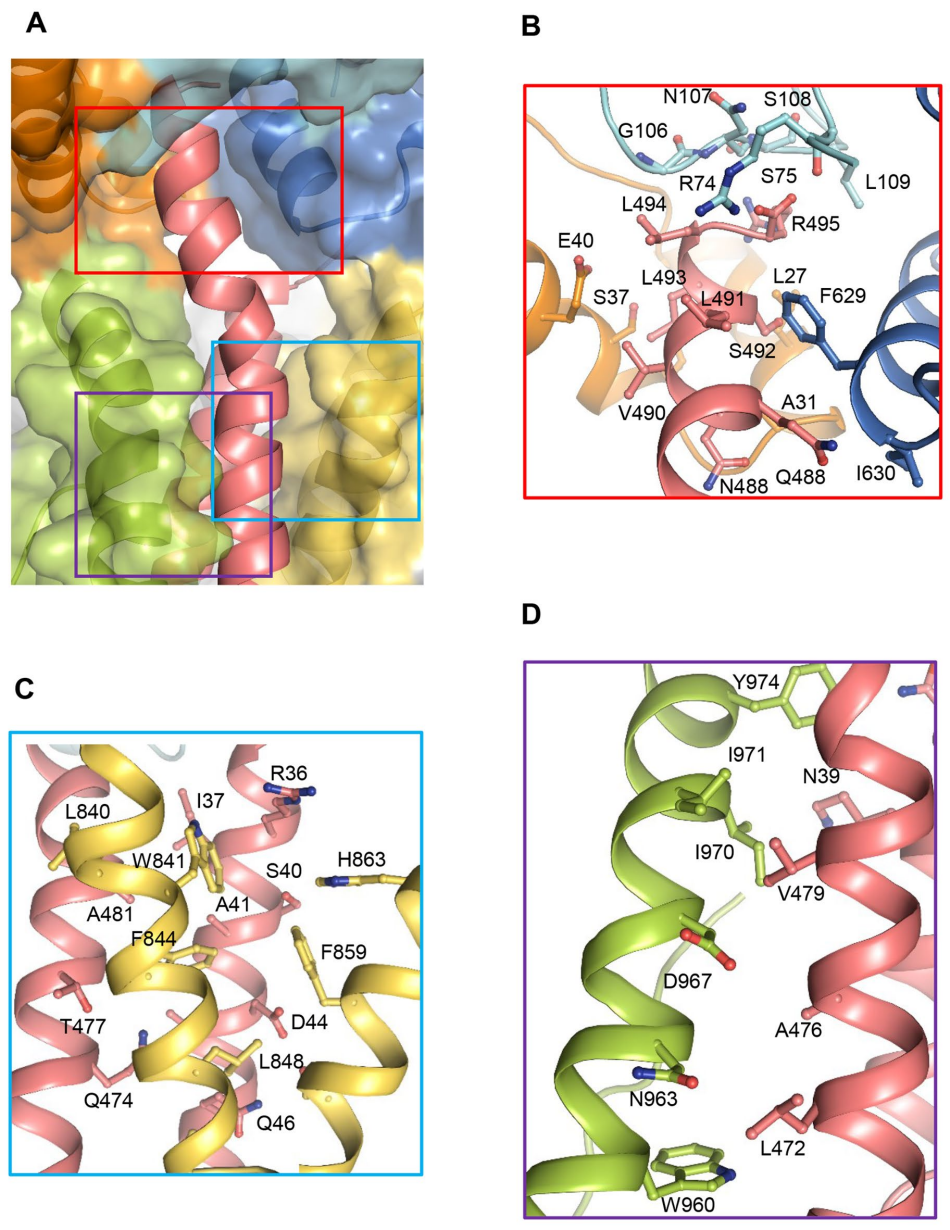
Recognition mechanism of Flic_D0_L_ by NAIP5. **(A)** A close-up view of the interaction of the C-terminal FliC_D0_L_ (cartoon) with NAIP5 (transparent surface). Detail interactions between FliC_D0_L_ and NAIP5 within the highlighted regions are shown in **B-D**. **(B)** Detailed interactions of the C-terminal side of FliC_D0_L_ with NAIP5 highlighted within the red square in **A**. **(C)** Detailed interactions of the central region of FliC_D0_L_ with NAIP5 highlighted within the blue square in **A**. **(D)** Detailed interactions of the C-terminal side of FliC_D0_L_ with NAIP5 ID highlighted within the purple square in **A**.

The extreme C-terminal side of FliC-C binds to a deep hydrophobic pocket formed by NTD, BIR1 and HD1 (Figures 3A and 4A). Four hydrophobic residues, L491, L493, L494 and V490 from this region of FliC-C contacts their neighboring residues from these three domains of NAIP5 (Figure 4B). Supporting this structural observation, simultaneous substitutions of L491, L493, L494 with alanines significantly compromised the activity of the C-terminal 35-residue peptide^21^ or full-length flagellin^12,13^ in activating NLRC4-mediated immune response. The central region of FliC-C is sandwiched between ID and one helix from HD2 of NAIP5 mainly via van der Waals and hydrophobic contacts (Figure 4A, 4C and 4D). Similar types of interactions are also important for mediating interaction of the helix portion of FliC-N with HD2 (Figure 4C) and binding of the N-terminal side of FliC-N to the surface groove formed by HD1 and HD2. The extensive contacts of HD2 with FliC_D0_L_ are consistent with previous data suggesting that the central NBD-associated domains of NAIP6 are crucial structural determinants for recognition of flagellin^31^. Structure-based sequence alignment indicate that the FliC_D0_L_-interacting residues of NAIP5 are highly conserved in NAIP6 but not in NAIP2 (Supplementary information, Figure S9), explaining the specific recognition of flagellin by NAIP5 or NAIP6^12,13^.

### Mutagenesis analysis of NAIP5 responsiveness to flagellin

**Figure 5:**
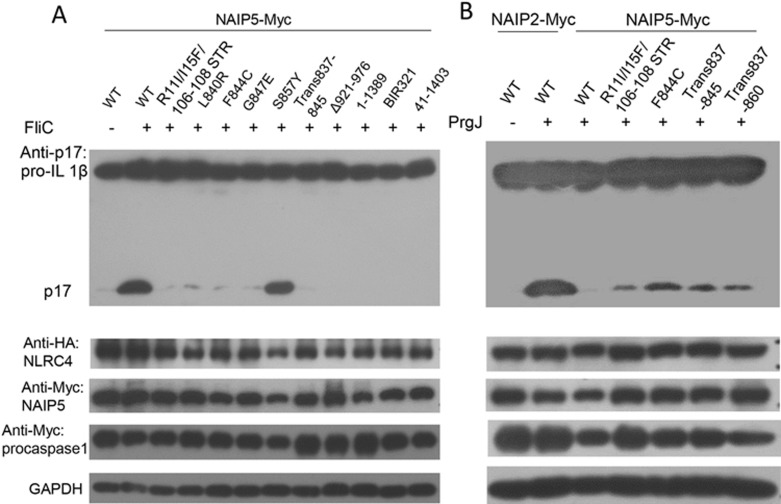
Mutagenesis analysis of NAIP5 responsiveness to flagellin. **(A)** Mutagenesis analyses of NAIP5 responsiveness to FliC. 293T cells were transfected with plasmids as indicated. 24 h after the transfection, the culture medium was supplemented with PA and LFn-FliC proteins with final concentration of 3 μg/mL. The cells were harvested and lysed, and the cleaved IL-1β was detected by anti-IL-1β immunoblotting analysis after 12 h. GAPDH was used as a loading control. Trans837-845: 837-845 residues of NAIP5 replaced with 880-888 residues of NAIP2. BIR321: exchange of BIR1(61-129) and BIR3(278-345) without changing linker region. **(B)** Mutagenesis analyses of NAIP5 responsiveness to PrgJ. The assays were performed as described in **A**.

**Figure 6:**
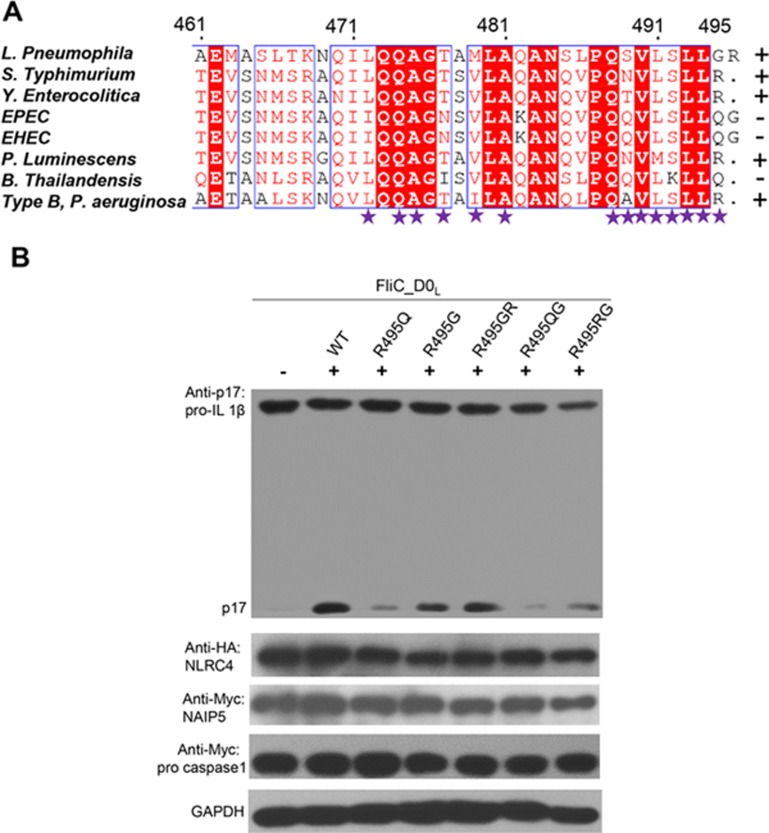
The last arginine residue of FliC_D0_L_ is an important epitope recognized by NAIP5. **(A)** Structure-based sequence alignment of the C-terminal sides of different bacterial flagellins. Flagellins with higher and lower activity of inducing NAIP5-NLRC4 inflammasome^13^ are indicated by “+” and “−”, respectively, in the last column. Residues interacting with NAIP5 from FliC_D0_L_ are indicated with stars at the bottom. Conserved and similar residues are in white and red, respectively. **(B)** Mutagenesis analyses of FliC_D0_L_ in NAIP5 activation. Wild-type and different FliC_D0_L_ mutants indicated were purified and used to assay their activity of inducing cleavage product of IL-1β. The assays were performed as described in Figure 5A.

We used the cell-based assay as previously described^13^ to further verify our structure. Amino acids from NAIP5 that are important for the interaction with FliC_D0_L_ were mutated and effects of these mutation on the activity of NAIP5 were monitored. As positive controls, FliC and FliC_D0_L_ strongly promoted the production of IL-1β in 293T cells when co-expressed with wild-type NAIP5, NLRC4 and procasepase-1 (Figures 5 and 6). In contrast, deletion of the N-terminal 40 residues of NAIP5 that cap FliC-C (Figure 3A) resulted in no detectable production of mature IL-1β induced by FliC. Furthermore, substitution of the three residues (106-108) from BIR1 that recognize the C-terminal side of FliC_D0_L_ together with other two non-conserved residues with their equivalents in NAIP2 greatly reduced FliC-induced maturation of IL-1β. These results agree with the observation that deletion of the BIR domains from NAIP5 resulted in loss of flagellin-induced activation of NAIP5-NLRC4 inflammasome^13^ and support an important role of BIR1 in dictating ligand specificity of NAIP5. Consistently, swapping of BIR1 and BIR3 of NAIP5 led to no detectable FliC-induced production of IL-1β. In further support of our structure, mutations of Leu840 and Gly847 from the helix in HD2 that simultaneously packs against the two helices of FliC_D0_L_ (Figure 4C) to their equivalents in NAIP1 also significantly reduced responsiveness of NAIP5 to FliC (Figure 5A). Similar observation was also made for the mutants of NAIP5 with Phe844 substituted by its corresponding Cys887 in NAIP2 and deletion of the C-terminal 14 residues, which contact the C-terminal side of FliC-C and stabilize the ID. In contrast, little effect on IL-1β maturation was observed for mutating S857, a residue that is solvent-exposed and does not interact with FliC_D0_L_. Strikingly, the NAIP5 mutant F844C was partially responsive to PrgJ in mediating the production of IL-1β (Figure 5B), further supporting an important role of this domain in mediating specific NAIP5 recognition of flagellins. However, further replacement of residues surrounding Phe844 with those in NAIP2 did not enhance responsiveness of the resulting NAIP5 mutants to PrgJ. Collectively, these results support our cryo-EM structure.

### Mechanism of differential flagellin recognition by NAIP5

Structure-based sequence alignment showed that the NAIP5-interacting residues of FliC_C are highly conserved among bacterial flagellin (Figure 6A). However, flagellins from different bacteria vary significantly in their NAIP5-interacting and NLRC4-inducing activities^13^. It is of interest to note that flagellins with higher activities have the arginine residue at their C-termini, whereas this residue is substituted with Gln or Gln-Gly in those with lower activities (Figure 6A). In the structure, the arginine residue of FliC_D0_L_ binds to a pocket of NAIP5 with a limited size (Figure 3B) and tightly packs against residues 106-109 from BIR1 (Figure 4B). These data suggest that the last arginine residue of flagellin may be important for its NAIP-NLRC4 inflammasome-inducing activity. In support of this possibility, mutation of the last residue Arg495 of FliC_D0_L_ to Gln significantly reduced its activity of inducing NAIP5 activation (Figure 6B). The activity was further decreased by addition of glycine to this FliC_D0_L_ mutant, likely because of steric effect caused by the limited size of the pocket recognizing the extreme C-terminal side of FliC_D0_L_. Consistently, introduction of Gly at the C-terminus of FliC_D0_L_ substantially compromised the peptide activity in inducing NAIP5 activation. However, the *Legionella pneumophila* flagellin with a glycine inserted before the last arginine residue has a similar activity to the *S. typhimurium* flagellin^13^. The reason for this might be that the C-terminal side of FliC_D0_L_ can be slightly kinked by introduction of the achiral glycine, thus allowing the C-terminal side to be accommodated by the NAIP5 pocket. Taken together, these results show that the last residue of flagellin is an important structural epitope recognized by NAIP5, although contributions from other positions to differential recognition of flagellins by NAIP5 are fully possible.

## Discussion

The data presented here showed that optimal recognition of FliC_D0_L_ involves multiple structural domains of NAIP5, although their contributions to interaction with the flagellin can vary. Binding of FliC_D0_L_ functions to stabilize the active conformation of NAIP5, indicating that NAIP5 as a seeding NLR needs a stabilized active conformation to ensure NLRC4 activation. A similar function can also be expected for the ligands of NAIP1 and its human homolog hNAIP, and NAIP2, which have been shown to play similar roles in ligand-induced assembly of NAIP-NLRC4 inflammasomes. Future structural studies are needed to investigate how these NAIPs specifically recognize their respective ligands. The observation that mutations of critical residues in HD2 of NAIP5 to their equivalents in NAIP1 significantly compromised FliC-induced activation of the NAIP5-NLRC4 inflammasome (Figure 5A) suggests that this structural domain may be also important for NAIP1 and hNAIP recognition of their ligands. In contrast with flagellin, cytochrome *c* is not involved in stabilizing the active conformation of APAF-1 in the APAF1 apoptosome^35,36^. It is noteworthy to mention that, however, assemblies of NAIP5-NLRC4 inflammasome and APAF-1 apoptosomes follow different mechanisms^14,15^. An active conformation-stabilizing role of FliC_D0_L_ in NAIP5 activation is in line with the observation that the constitutively active plant NLR L6 displayed a higher ligand binding affinity than wild-type protein^37^. In contrast with those in the NAIP5-NLRC4 inflammasome^14,15,16^, NLRC4 from the dimeric NAIP5-NLRC4^M^ complex is much less defined, indicating that oligomerization of NLRC4 is important for stabilization of its active conformation. These results appear to suggest that stabilization of the active conformation of an NLR protein can play an important role in its activation. But more studies are needed to determine whether ligand recognition or perception by other NLRs has a similar function to flagellin binding to NAIP5.

Modeling studies showed that the inactive NAIP5 has a similar conformation to that of the inactive NLRC4^31,38^. Then how is flagellin initially recognized by an inactive NAIP5? Structural comparison between the active NAIP5 and a modeled inactive NAIP5 (Supplementary information, Figure S10) suggested that the FliC_D0_L_ binding pocket formed by BIR1 and HD1 is an attractive site for initial recognition of the extreme C-terminal side of FliC_D0_L_, because other FliC-D0_L_ binding sites are largely occluded by the positioning of LRR in the inactive NAIP5. This can afford an explanation for the ability of NAIP5 to differentiate bacterial flagellins with subtle differences in their C-terminal sides. However, we cannot exclude the possibility that inactive NAIP5 assumes a structure strikingly different from the predicted one. Nonetheless, nearly complete burial of FliC_D0_L_ indicates that the conformation of inactive NAIP5 should be different from its active one. Thus, FliC_D0_L_ binding is expected to trigger structural re-organization to the active conformation of NAIP5 for full interaction with flagellin. Conformational selection, proposed for self-activation of NLRC4^14^, may also be involved in initial flagellin binding to NAIP5. In this case, NAIP5 may adopt a metastable active state that exists in an equilibrium with its more stable inactive state. Stabilization by flagellin binding can shift the equilibrium toward the active state of NAIP5.

Structural studies showed that dATP/ATP acts to stabilize the active conformation of APAF-1 via the γ-phosphate of the bound dATP/ATP, supporting an essential role of dATP/ATP in assembly of the APAF-1 apoptosome^35,36^. However, the γ-phosphate of the NAIP5-bound ATP does not interact with other domains than the NBD and HD1, indicating that ATP makes no direct contribution to the stabilization of the active conformation of NAIP5. Mutations^32^ of P-loop showed that nucleotide binding of NAIP5 is not essential for formation of NAIP5-NLRC4 inflammasome. Our previous studies^14,38^ suggested that ATP binding may be a consequence of ligand-induced conformation changes in NLRC4. Collectively, these data suggest that a role of the bound ATP in NLR activation might be associated with whether it acts to stabilize the ligand-induced active conformation of the NLR protein^16^.

Our structural and biochemical data showed that the last arginine residue from the tested flagellins with higher NLRC4 inflammasome-inducing activity^13^ is an important epitope recognized by NAIP5. Mutation of this residue in *S. typhimurium* flagellin has been shown to impair bacterial mobility^39^, suggesting a correlation between the functional significance of the arginine residue as a critical moiety of PAMP for bacteria and its recognition by NAIP5. A similar correlation has been demonstrated for flagellin recognition by TLR5^39,40^. Some bacterial flagellins possess different residues at this position and display much lower NLRC4 inflammasome-inducing activity^13^, suggesting that these alterations might be a bacterial strategy to evade host immune recognition by NAIP5. On the other hand, at least for *EPEC* and *Burkholderia thailandensis* carrying such altered flagellins, flagellin-independent caspase-1 activation has been demonstrated during infection^13^, probably reflecting an evolutionary arms race between bacteria and their hosts.

## Materials and Methods

### Plasmids and antibodies

Full-length *NAIP5* and *NAIP2* genes were obtained by DNA synthesis. Mouse *NLRC4*, *pro IL-1*β and *pro caspase-1* were amplified from reverse-transcribed mouse cDNAs. Full-length *PrgJ* and *FliC* genes were amplified from *S. typhimurium* genomic DNA. NLRC4-R288A-L435D-1 008-1 012^DDYD−AAAA^(NLRC4^M^), all NAIP5 and flagellin mutations were generated by standard molecular biology procedures. FliC_D0_L_ was designed as described before^32^. All constructs were verified by sequencing.

Antibody used: anti-Myc (cw0299, CWBIO), anti-HA (cw0092, CWBIO), anti-IL-1β (GTX74034, GeneTex) and anti-GAPDH (cw0100, CWBIO).

### Recombinant protein expression and purification

A heterodimeric complex between NAIP5 and NLRC4^M^ induced by FliC_D0_L_ was purified from insect cells as described previously^14^). Briefly, the related genes were cloned into modified pFastBac vectors containing cleavable N-terminal GST or N-terminal 6× His-SUMO tag and co-expressed in sf21 insect cells (Invitrogen). Sf21 cells were grown in SF900 (GIBICO) medium by shaking at 120 rpm at 28 °C until the density reached 2.0 × 10^6^/mL. One liter of cells (2.0 × 10^6^/mL) were infected with 22 mL of recombinant baculovirus. Cells were harvested after 48 h of infection, re-suspended in the buffer containing 25 mM Tris (pH 8.0), 150 mM NaCl and 1 mM PMSF, and lysed by sonication before centrifugation. The supernatant was flowed through glutathione sepharose 4B beads (Invitrogen). The bound proteins were digested with PreScission protease (GE Healthcare) to remove the GST tag and further purified by size exclusion chromatography (Hiload 16/60 Superdex 200 prep grade, GE Healthcare) in a buffer containing 10 mM Tris (pH 8.0) and 150 mM NaCl.

PrgJ, FliC, and FliC_D0_L_ mutants used in 293 assays were fused with N-terminal 1-263 aa of lethal factor (LF_n_) and cloned to pET15b vector. *Escherichia coli* BL21(DE3) stains transformed with the expression plasmids were grown in LB medium and induced overnight at 18 °C with 0.6 mM IPTG after OD_600_ reached 0.7. Bacteria were harvested, re-suspended and lysed in the buffer containing 25 mM Tris (pH 8.0) and 150 mM NaCl. The soluble fraction was purified by Ni-NTA (Novagen) and further purified by size exclusion chromatography (Superdex 200, GE Healthcare) in a buffer containing 10 mM Tris (pH 8.0) and 150 mM NaCl.

### Analysis of NLRC4 inflammasome in 293T cells

*mNAIP5*, *mNAIP2*, mouse *pro IL-1β*, *pro caspase1* and *NAIP5* mutations were cloned to pcDNA3.1 vector. 293T cells were seeded to six-well plate 12 h before transfection. The complex of plasmids containing 1 μg *pro IL-1β*, 50 ng *pro caspase 1*, 50 ng *NLRC4*, *NAIP5* and 2 μl vigofect reagent (Vigorous) per well were added to the culture supernatant. After 24 h of transfection, PA and LF_n_ fused proteins were added to the culture medium with a final concentration of 3 μg/mL. After another 12 h, the transfected cells were harvested in the lysis buffer containing 25 mM Tris 7.4, 150 mM NaCl, 1% Triton X-100 and 1× protease inhibitor cocktail (Thermo Scientific). The cleaved IL-1β was detected by anti-IL-1β immunoblotting analysis. All the reconstitution experiments were performed at least three times.

### Cryo-EM sample preparation and data collection

The purified FliC_D0_L_-NAIP5-NLRC4^M^ complex was concentrated to ∼2 mg/mL. Then aliquots of 4 μl of this sample were applied to the glow-discharged Quantifoil R1.2/1.3 holey carbon grids (Quantifoil Micro Tools GmbH), blotted for 2 s and plunge-frozen by FEI Vitrobot Mark IV. Grids were examined using an FEI Titan Krios operated at 300 kV, and images were recorded using a K2 Summit direct electron detector (Gatan) in super-resolution mode, at a nominal magnification of 22 500, and with the defocus ranging from −1.5 to −3.0 μm. Images were collected under low-dose condition in a semiautomatic manner using UCSF-Image4^41^. The dose rate on the camera was set to be 8.2 electrons per pixel per second. For each micrograph stack, a total of 32 frames were collected with an exposure time of 8 s, leading to a total accumulated dose of 50 electrons per Å^2^ on the specimen.

### Cryo-EM image processing and refinement

The collected original micrographs stacks were aligned and summed using whole image-motion correction^41^, and binned two-fold, resulting in a pixel size of 1.30654 Å per pixel. The defocus values of micrographs were estimated by CTFFIND3^42^. The collected particles were picked by EMAN2^43^ and RELION 1.4^44,45^. 2D and 3D classifications and refinements were performed using RELION 1.4^44,45^.

A total of 123 876 particles were first boxed from 525 micrographs of the FliC_D0_L_-NAIP5-NLRC4^M^ complex using e2boxer.py in EMAN2^43^, respectively. Then, the boxed particles were extracted and reference-free 2D class averaging was performed. The generated 2D class averages were used as the templates for the subsequent autopicking of particles of Naip5-Nlrc4 complex using Relion 1.4^44,45^. A total of 2 192 107 particles of the NAIP5-NLRC4 complex were automatically picked out from 2 864 micrographs, respectively, and 2D classified using Relion 1.4^44,45^. About 1 663 317 particles were empirically selected from 2D classifications using and auto-refined against model from EMDB (ID: 3143) as an initial model. The obtained reconstructions low-pass filtered to 60 Å were then used as the references of the 3D classifications. All of the 1 663 317 particles were divided into 10 classes. The two most homogeneous classes of 245 315 particles and 381 293 particles were subjected to auto-refinement without any symmetry imposed and resulted in reconstructions at an overall resolution of 6.53 and 7.90 Å, respectively, based on the gold-standard FSC 0.143 criterion^46^. The original image stacks were then aligned and summed using whole image-motion correction2^47^, and binned two-fold. All the particles above had been replaced by particles from new summed micrographs from image-motion correction2, with keeping the alignment parameters already obtained from previous auto-refinement using RELION 1.4^44,45^. All of the new 245 315 and 381 293 particles were subjected to auto-refinement with local search methods using RELION 1.4^44,45^, and at this stage the resolution of these two classes of particles were refined to 4.93 and 4.58 Å, respectively. A soft mask was applied around the rigid part of NAIP5, which further improved resolution to, 4.42 and 4.51 Å, respectively. All the particles of the two classes were then merged together for auto-refinement with the soft mask imposed around the rigid part of NAIP5 and resulted in a reconstruction at a resolution of 4.28 Å. Additional cycles of 3D classifications and refinements did not further improve the overall resolution of the maps. Local resolution was estimated using ResMap^48^.

### Model building and structure refinement

To build the NAIP5 model, the previously reported activated NLRC4 structure and baculoviral IAP repeat-containing protein 7 (PDB 1TW6, chain A) were used as the initial models. The initial NLRC4 model, in which the conserved residues in NLRC4 were retained and the non-conserved residues were substituted with alanine residues using CHAINSAW^49^. We individually separated the NBD, HD1, WHD and LRR domains of NLRC4 and rigid body fitted these domains into the EM map in COOT^50^. The chain A of baculoviral IAP repeat-containing protein 7 was fitted into the electron density from the BIR1 and BIR2 domains. Residues 921-980 from the ID of NAIP5 were manually built into the 3D EM density in COOT. *De novo* model building was performed for FliC_D0_L_ by manually docking two alpha helices into the electron density. Residue assignment of FliC_D0_L_ was guided by its last arginine and other bulky residues.

The molecular dynamics flexible fitting (MDFF)^51^ method was used to flexibly fit the atomic structure into the density map in VMD after model building in COOT. The model was finally refined against the map at overall 4.28 Å using phenix.real_space_refine application in PHENIX^52^ in real space with secondary structure and geometry restraints to prevent structure over-fitting. Residues with Ramachandran outliers were further manually adjusted in COOT. The coordinates of the final model (FliC_D0_L_-NAIP5-NLRC4^M^ complex) were randomly displaced by 0.5 Å using the Phenix (PDB Tools) to remove potential model bias. The displaced model was then refined against one of the half maps (produced from a half set of all particles during refinement by RELION). FSC curves were calculated between the resulting model and half1 map (model versus half1 map, FSCwork, that is, used for refinement), the resulting model and half2 map (model versus half2 map, FSCfree, that is, not used for refinement) and the resulting model and the final density map (model versus summer map) from all particles. The lack of significant separation between work and free FSC curves suggested that the models were not over-fitted.

## Author Contributions

JC and S-FS conceived, designed the project and wrote the manuscript. JC and S-FS supervised the project. ZH made initial contribution to the project. XY and GL purified the proteins for EM and performed 293 assays. XY and FY performed cryo-EM sample preparation and data collection. FY calculated the cryo-EM map and WW built the atomic model. All authors contributed to project discussion and manuscript preparation.

## Competing Financial Interests

The authors declare no competing financial interests.

## Supplementary Information

Supplementary information, Figure S1 Purification of the FliC_D0_L_-NAIP5-NLRC4^M^ complex

Supplementary information, Figure S2 Structure determination of the FliC_D0_L_-NAIP5-NLRC4^M^ complex by Cryo-EM

Supplementary information, Figure S3 Cryo-EM analysis of the FliC_D0_L_-NAIP5-NLRC4^M^ complex

Supplementary information, Figure S4 Interaction of ATP with the FliC_D0_L_-bound NAIP5

Supplementary information, Figure S5 Representative EM density maps for different structural domains from the FliC_D0_L_-NAIP5-NLRC4^M^ complex

Supplementary information, Figure S6 Structural superposition of NAIP5-NLRC4 with NLRC4 inflammasome

Supplementary information, Figure S7 BIR3 is likely involved in closure of the wheel-like structure of NAIP5-NLRC4 inflammasome

Supplementary information, Figure S8 Structural comparison of BIR1, BIR2 of NAIP5 and BIR3 of XIAP.

Supplementary information, Figure S9 Structure-based sequence alignment of mouse NAIPs.

Supplementary information, Figure S10 Structural comparison between the active NAIP5 and a modeled inactive NAIP5

Supplementary information, Table S1 Data collection and model statistics.

## References

[bib1] Schroder K, Tschopp J. The inflammasomes. Cell 2010; 140:821–832. 20303873 10.1016/j.cell.2010.01.040

[bib2] Davis BK, Wen H, Ting JP. The inflammasome NLRs in immunity, inflammation, and associated diseases. Annu Rev Immunol 2011; 29:707–735. 21219188 10.1146/annurev-immunol-031210-101405 PMC4067317

[bib3] Franchi L, Munoz-Planillo R, Nunez G. Sensing and reacting to microbes through the inflammasomes. Nat Immunol 2012; 13:325–332. 22430785 10.1038/ni.2231 PMC3449002

[bib4] Rathinam VA, Vanaja SK, Fitzgerald KA. Regulation of inflammasome signaling. Nat Immunol 2012; 13:333–342. 22430786 10.1038/ni.2237 PMC3523703

[bib5] Jones JD, Vance RE, Dangl JL. Intracellular innate immune surveillance devices in plants and animals. Science 2016; 354: pii: aaf6395. 27934708 10.1126/science.aaf6395

[bib6] von Moltke J, Ayres JS, Kofoed EM, Chavarria-Smith J, Vance RE. Recognition of bacteria by inflammasomes. Annu Rev Immunol 2013; 31:73–106. 23215645 10.1146/annurev-immunol-032712-095944

[bib7] Martinon F, Burns K, Tschopp J. The inflammasome: a molecular platform triggering activation of inflammatory caspases and processing of proIL-beta. Mol Cell 2002; 10:417–426. 12191486 10.1016/s1097-2765(02)00599-3

[bib8] Vance RE. The NAIP/NLRC4 inflammasomes. Curr Opin Immunol 2015; 32:84–89. 25621709 10.1016/j.coi.2015.01.010 PMC4336817

[bib9] Shi J, Zhao Y, Wang K, et al. Cleavage of GSDMD by inflammatory caspases determines pyroptotic cell death. Nature 2015; 526:660–665. 26375003 10.1038/nature15514

[bib10] Kayagaki N, Stowe IB, Lee BL, et al. Caspase-11 cleaves gasdermin D for non-canonical inflammasome signalling. Nature 2015; 526:666–671. 26375259 10.1038/nature15541

[bib11] He WT, Wan H, Hu L, et al. Gasdermin D is an executor of pyroptosis and required for interleukin-1beta secretion. Cell Res 2015; 25:1285–1298. 26611636 10.1038/cr.2015.139 PMC4670995

[bib12] Kofoed EM, Vance RE. Innate immune recognition of bacterial ligands by NAIPs determines inflammasome specificity. Nature 2011; 477:592–595. 21874021 10.1038/nature10394 PMC3184209

[bib13] Zhao Y, Yang J, Shi J, et al. The NLRC4 inflammasome receptors for bacterial flagellin and type III secretion apparatus. Nature 2011; 477:596–600. 21918512 10.1038/nature10510

[bib14] Hu Z, Zhou Q, Zhang C, et al. Structural and biochemical basis for induced self-propagation of NLRC4. Science 2015; 350:399–404. 26449475 10.1126/science.aac5489

[bib15] Zhang L, Chen S, Ruan J, et al. Cryo-EM structure of the activated NAIP2-NLRC4 inflammasome reveals nucleated polymerization. Science 2015; 350:404–409. 26449474 10.1126/science.aac5789 PMC4640189

[bib16] Diebolder CA, Halff EF, Koster AJ, Huizinga EG, Koning RI. Cryoelectron tomography of the NAIP5/NLRC4 inflammasome: implications for NLR activation. Structure 2015; 23:2349–2357. 26585513 10.1016/j.str.2015.10.001

[bib17] Zhao Y, Shao F. The NAIP-NLRC4 inflammasome in innate immune detection of bacterial flagellin and type III secretion apparatus. Immunol Rev 2015; 265:85–102. 25879286 10.1111/imr.12293

[bib18] Miller LK. An exegesis of IAPs: salvation and surprises from BIR motifs. Trends Cell Biol 1999; 9:323–328. 10407412 10.1016/s0962-8924(99)01609-8

[bib19] Franchi L, Amer A, Body-Malapel M, et al. Cytosolic flagellin requires Ipaf for activation of caspase-1 and interleukin 1beta in salmonella-infected macrophages. Nat Immunol 2006; 7:576–582. 16648852 10.1038/ni1346

[bib20] Miao EA, Alpuche-Aranda CM, Dors M, et al. Cytoplasmic flagellin activates caspase-1 and secretion of interleukin 1beta via Ipaf. Nat Immunol 2006; 7:569–575. 16648853 10.1038/ni1344

[bib21] Lightfield KL, Persson J, Brubaker SW, et al. Critical function for Naip5 in inflammasome activation by a conserved carboxy-terminal domain of flagellin. Nat Immunol 2008; 9:1171–1178. 18724372 10.1038/ni.1646 PMC2614210

[bib22] Molofsky AB, Byrne BG, Whitfield NN, et al. Cytosolic recognition of flagellin by mouse macrophages restricts *Legionella pneumophila* infection. J Exp Med 2006; 203:1093–1104. 16606669 10.1084/jem.20051659 PMC1584282

[bib23] Sutterwala FS, Mijares LA, Li L, Ogura Y, Kazmierczak BI, Flavell RA. Immune recognition of *Pseudomonas aeruginosa* mediated by the IPAF/NLRC4 inflammasome. J Exp Med 2007; 204:3235–3245. 18070936 10.1084/jem.20071239 PMC2150987

[bib24] Miao EA, Mao DP, Yudkovsky N, et al. Innate immune detection of the type III secretion apparatus through the NLRC4 inflammasome. Proc Natl Acad Sci USA 2010; 107:3076–3080. 20133635 10.1073/pnas.0913087107 PMC2840275

[bib25] Ren T, Zamboni DS, Roy CR, Dietrich WF, Vance RE. Flagellin-deficient Legionella mutants evade caspase-1- and Naip5-mediated macrophage immunity. PLoS Pathog 2006; 2:e18. 16552444 10.1371/journal.ppat.0020018 PMC1401497

[bib26] Rayamajhi M, Zak DE, Chavarria-Smith J, Vance RE, Miao EA. Cutting edge: Mouse NAIP1 detects the type III secretion system needle protein. J Immunol 2013; 191:3986–3989. 24043898 10.4049/jimmunol.1301549 PMC3819181

[bib27] Yang J, Zhao Y, Shi J, Shao F. Human NAIP and mouse NAIP1 recognize bacterial type III secretion needle protein for inflammasome activation. Proc Natl Acad Sci USA 2013; 110:14408–14413. 23940371 10.1073/pnas.1306376110 PMC3761597

[bib28] Rauch I, Tenthorey JL, Nichols RD, et al. NAIP proteins are required for cytosolic detection of specific bacterial ligands *in vivo*. J Exp Med 2016; 213:657–665. 27045008 10.1084/jem.20151809 PMC4854734

[bib29] Zhao Y, Shi J, Shi X, Wang Y, Wang F, Shao F. Genetic functions of the NAIP family of inflammasome receptors for bacterial ligands in mice. J Exp Med 2016; 213:647–656. 27114610 10.1084/jem.20160006 PMC4854738

[bib30] Rauch I, Deets KA, Ji DX, et al. NAIP-NLRC4 inflammasomes coordinate intestinal epithelial cell expulsion with eicosanoid and IL-18 release via activation of caspase-1 and -8. Immunity 2017; 46:649–659. 28410991 10.1016/j.immuni.2017.03.016 PMC5476318

[bib31] Tenthorey JL, Kofoed EM, Daugherty MD, Malik HS, Vance RE. Molecular basis for specific recognition of bacterial ligands by NAIP/NLRC4 inflammasomes. Mol Cell 2014; 54:17–29. 24657167 10.1016/j.molcel.2014.02.018 PMC3988258

[bib32] Halff EF, Diebolder CA, Versteeg M, Schouten A, Brondijk TH, Huizinga EG. Formation and structure of a NAIP5-NLRC4 inflammasome induced by direct interactions with conserved N- and C-terminal regions of flagellin. J Biol Chem 2012; 287:38460–38472. 23012363 10.1074/jbc.M112.393512 PMC3493891

[bib33] Wu G, Chai J, Suber TL, et al. Structural basis of IAP recognition by Smac/DIABLO. Nature 2000; 408:1008–1012. 11140638 10.1038/35050012

[bib34] Maki-Yonekura S, Yonekura K, Namba K. Conformational change of flagellin for polymorphic supercoiling of the flagellar filament. Nat Struct Mol Biol 2010; 17:417–422. 20228803 10.1038/nsmb.1774

[bib35] Zhou M, Li Y, Hu Q, et al. Atomic structure of the apoptosome: mechanism of cytochrome c- and dATP-mediated activation of Apaf-1. Genes Dev 2015; 29:2349–2361. 26543158 10.1101/gad.272278.115 PMC4691890

[bib36] Cheng TC, Hong C, Akey IV, Yuan S, Akey CW. A near atomic structure of the active human apoptosome. eLife 2016; 5: pii: e17755. 27697150 10.7554/eLife.17755 PMC5050015

[bib37] Bernoux M, Burdett H, Williams SJ, et al. Comparative analysis of the Flax immune receptors L6 and L7 suggests an equilibrium-based switch activation model. Plant Cell 2016; 28:146–159. 26744216 10.1105/tpc.15.00303 PMC4746675

[bib38] Hu Z, Yan C, Liu P, et al. Crystal structure of NLRC4 reveals its autoinhibition mechanism. Science 2013; 341:172–175. 23765277 10.1126/science.1236381

[bib39] Forstneric V, Ivicak-Kocjan K, Plaper T, Jerala R, Bencina M. The role of the C-terminal D0 domain of flagellin in activation of Toll like receptor 5. PLoS Pathog 2017; 13:e1006574. 28827825 10.1371/journal.ppat.1006574 PMC5578693

[bib40] Andersen-Nissen E, Smith KD, Strobe KL, et al. Evasion of Toll-like receptor 5 by flagellated bacteria. Proc Natl Acad Sci USA 2005; 102:9247–9252. 15956202 10.1073/pnas.0502040102 PMC1166605

[bib41] Li X, Mooney P, Zheng S, et al. Electron counting and beam-induced motion correction enable near-atomic-resolution single-particle cryo-EM. Nat Methods 2013; 10:584–590. 23644547 10.1038/nmeth.2472 PMC3684049

[bib42] Mindell JA, Grigorieff N. Accurate determination of local defocus and specimen tilt in electron microscopy. J Struct Biol 2003; 142:334–347. 12781660 10.1016/s1047-8477(03)00069-8

[bib43] Tang G, Peng L, Baldwin PR, et al. EMAN2: an extensible image processing suite for electron microscopy. J Struct Biol 2007; 157:38–46. 16859925 10.1016/j.jsb.2006.05.009

[bib44] Scheres SH. A Bayesian view on cryo-EM structure determination. J Mol Biol 2012; 415:406–418. 22100448 10.1016/j.jmb.2011.11.010 PMC3314964

[bib45] Scheres SH. RELION: implementation of a Bayesian approach to cryo-EM structure determination. J Struct Biol 2012; 180:519–530. 23000701 10.1016/j.jsb.2012.09.006 PMC3690530

[bib46] Scheres SH, Chen S. Prevention of overfitting in cryo-EM structure determination. Nat Methods 2012; 9:853–854. 22842542 10.1038/nmeth.2115 PMC4912033

[bib47] Zheng SQ, Palovcak E, Armache JP, Verba KA, Cheng Y, Agard DA. MotionCor2: anisotropic correction of beam-induced motion for improved cryo-electron microscopy. Nat Methods 2017; 14:331–332. 28250466 10.1038/nmeth.4193 PMC5494038

[bib48] Kucukelbir A, Sigworth FJ, Tagare HD. Quantifying the local resolution of cryo-EM density maps. Nat Methods 2014; 11:63–65. 24213166 10.1038/nmeth.2727 PMC3903095

[bib49] Stein N. CHAINSAW: a program for mutating pdb files used as templates in molecular replacement. J Appl Crystallogr 2010; 41:641–643.

[bib50] Emsley P, Lohkamp B, Scott WG, Cowtan K. Features and development of Coot. Acta Crystallogr D Biol Crystallogr 2010; 66:486–501. 20383002 10.1107/S0907444910007493 PMC2852313

[bib51] Trabuco LG, Villa E, Mitra K, Frank J, Schulten K. Flexible fitting of atomic structures into electron microscopy maps using molecular dynamics. Structure 2008; 16:673–683. 18462672 10.1016/j.str.2008.03.005 PMC2430731

[bib52] Adams PD, Afonine PV, Bunkoczi G, et al. PHENIX: a comprehensive Python-based system for macromolecular structure solution. Acta Crystallogr D Biol Crystallogr 2010; 66:213–221. 20124702 10.1107/S0907444909052925 PMC2815670

