# Supplementary material for: Structural basis for specific flagellin recognition by the NLR protein NAIP5
Source: Cell Res. 2017 Nov 28;28(1):35–47. doi: 10.1038/cr.2017.148 (PMC5752844; doi:10.1038/cr.2017.148)
Supplement: Supplementary information, Figure S1 — Purification of the FliC_D0L-NAIP5-NLRC4M complex [file cr2017148x1.pdf]

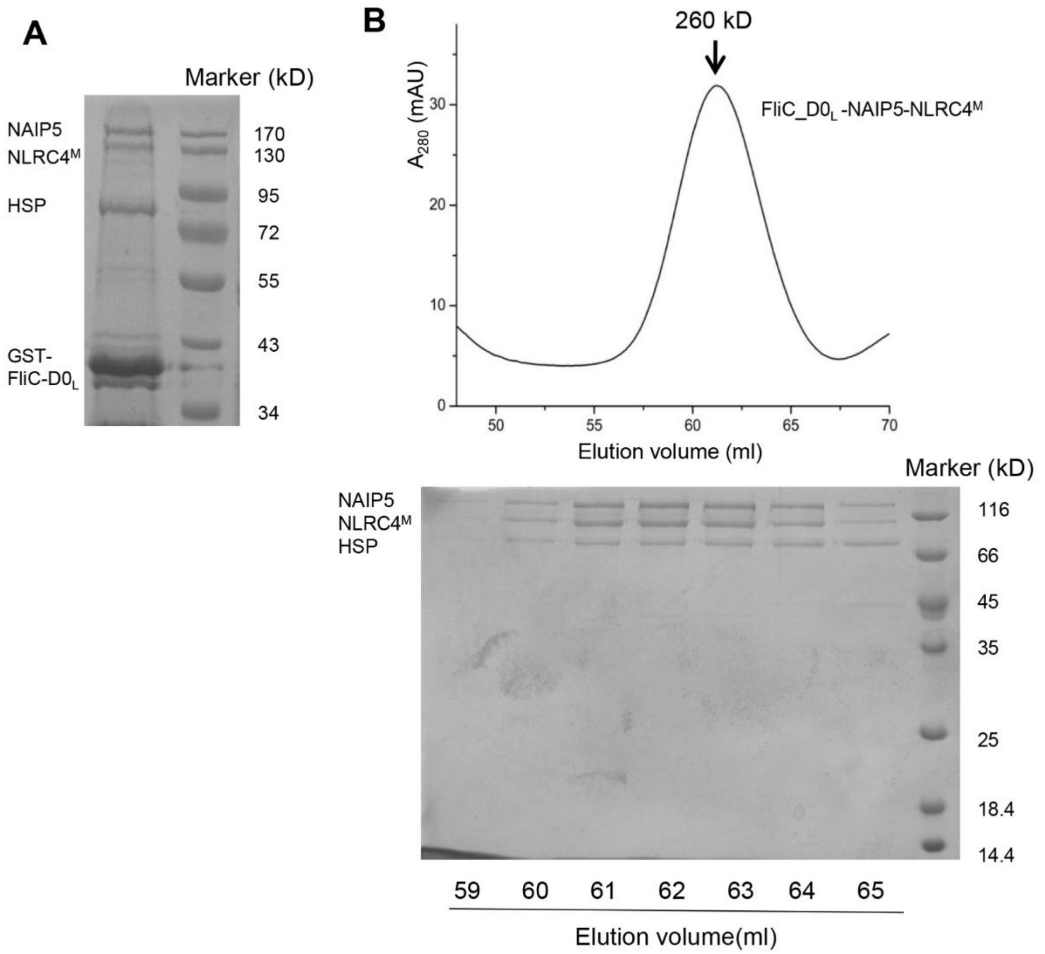
A
Marker (kD)
NAIP5
NLRC4M
HSP
GST-
FliC-D0L
170
130
95
72
55
43
34
B
260 kD
FliC_D0L-NAIP5-NLRC4M
A280 (mAU)
0
10
20
30
50
55
60
65
70
Elution volume (ml)
Marker (kD)
NAIP5
NLRC4M
HSP
116
66
45
35
25
18.4
14.4
59
60
61
62
63
64
65
Elution volume(ml)

Supplementary information, Figure S1. Purification of the FliC_D0$_L$ -NAIP5-NLRC4^M complex

(A) Affinity purification of the FliC_D0$_L$ -NAIP5-NLRC4^M complex. GST-FliC_D0$_L$, NAIP5 and NLRC4^M were co-expressed in insect cells. Cells were lysated and flowed through GS4B resins. After extensive washing, the bound proteins were eluted with the buffer containing 15 mM GSH. The eluent was detected by SDS-PAGE followed by Coomassie blue staining. Molecular weights of marker proteins are labeled on the right side.

(B) Gel filtration profile (top) of the FliC_D0$_L$ -NAIP5-NLRC4^M complex. After removal of GST by prescission protease cleavage, the proteins purified in (A) were subjected to gel filtration for further purification. Peak fractions were visualized by SDS-PAGE followed by Coomassie blue staining (bottom). The band of FLIC_D0$_L$ was too shallow to be seen.
