# Supplementary material for: Structural basis for specific flagellin recognition by the NLR protein NAIP5
Source: Cell Res. 2017 Nov 28;28(1):35–47. doi: 10.1038/cr.2017.148 (PMC5752844; doi:10.1038/cr.2017.148)
Supplement: Supplementary information, Figure S2 — Structure determination of the FliC_D0L-NAIP5-NLRC4M complex by Cryo-EM [file cr2017148x2.pdf]

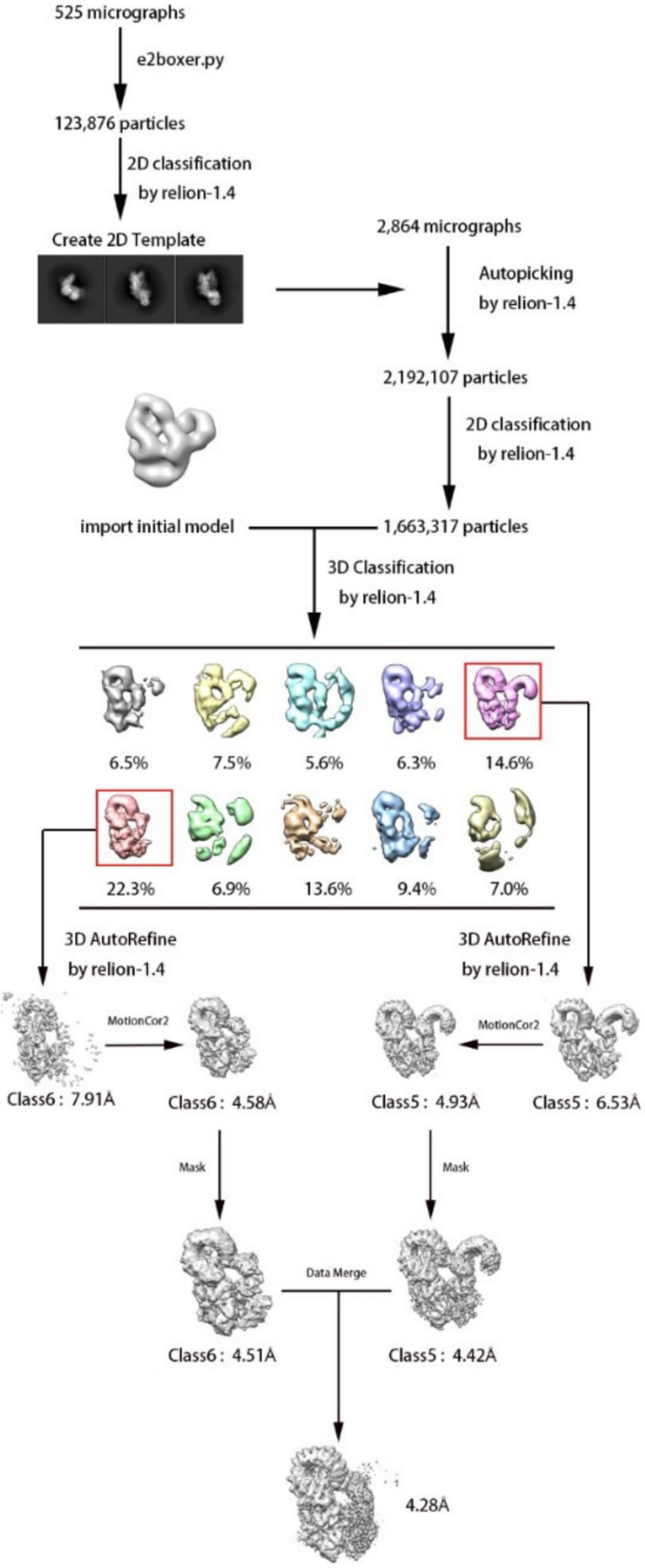
525 micrographs
e2boxer.py
123,876 particles
2D classification
by relion-1.4
Create 2D Template
2,864 micrographs
Autopicking
by relion-1.4
2,192,107 particles
2D classification
by relion-1.4
import initial model
1,663,317 particles
3D Classification
by relion-1.4
6.5%
7.5%
5.6%
6.3%
14.6%
22.3%
6.9%
13.6%
9.4%
7.0%
3D AutoRefine
by relion-1.4
3D AutoRefine
by relion-1.4
MotionCor2
MotionCor2
Class6 : 7.91Å
Class6 : 4.58Å
Class5 : 4.93Å
Class5 : 6.53Å
Mask
Mask
Data Merge
Class6 : 4.51Å
Class5 : 4.42Å
4.28Å

Supplementary information, Figure S2. Structure determination of the FliC_D0$_L$ -NAIP5-NLRC4^M complex by Cryo-EM

A flowchart of Cryo-EM data processing and 3D reconstruction. Please refer to Materials and Methods for details.
