# Supplementary material for: Structural basis for specific flagellin recognition by the NLR protein NAIP5
Source: Cell Res. 2017 Nov 28;28(1):35–47. doi: 10.1038/cr.2017.148 (PMC5752844; doi:10.1038/cr.2017.148)
Supplement: Supplementary information, Figure S3 — Cryo-EM analysis of the FliC_D0L-NAIP5-NLRC4M complex [file cr2017148x3.pdf]

A

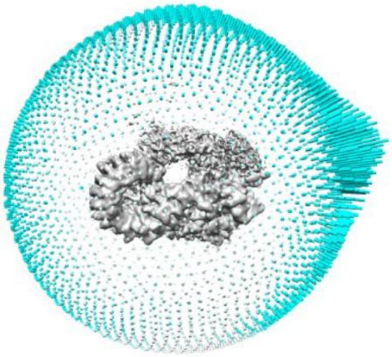

B

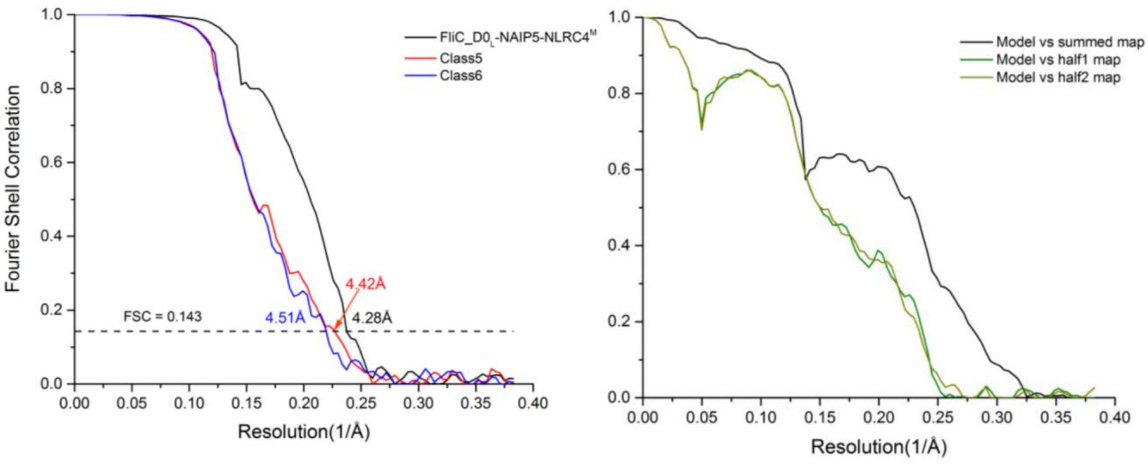

Fourier Shell Correlation
Resolution(1/Å)
FliC_D0L-NAIP5-NLRC4M
Class5
Class6
FSC = 0.143
4.42Å
4.51Å
4.28Å
Model vs summed map
Model vs half1 map
Model vs half2 map

C

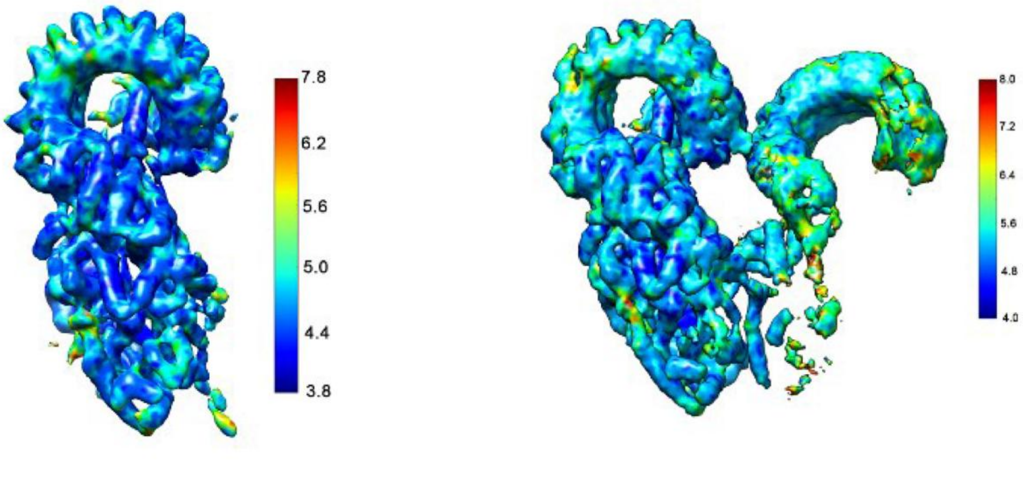

7.8
6.2
5.6
5.0
4.4
3.8
8.0
7.2
6.4
5.6
4.8
4.0

Supplementary information, Figure S3. Cryo-EM analysis of the FliC_D0$_L$ -NAIP5-NLRC4^M complex

(A) Euler angle distributions of particles used for the final reconstruction of the FliC_D0$_L$-NAIP5-NLRC4^M complex. Each cylinder represents one view and its size is proportional to the number of particles represented.

(B) Left: Gold-standard FSC curves of the final reconstructions of the FliC_D0$_L$ -NAIP5-NLRC4^M complex with different conformations. Right: FSC curves for the cross-validation of the atomic models of the FliC_D0$_L$ -NAIP5-NLRC4^M complex.

(C) The final EM density map (left) and the Class5 EM density map (right) of the FliC_D0$_L$ -NAIP5-NLRC4^M complex with color coded to show the local resolution as estimated by ResMap.
