# Supplementary material for: Structural basis for specific flagellin recognition by the NLR protein NAIP5
Source: Cell Res. 2017 Nov 28;28(1):35–47. doi: 10.1038/cr.2017.148 (PMC5752844; doi:10.1038/cr.2017.148)
Supplement: Supplementary information, Figure S4 — Interaction of ATP with the FliC_D0L-bound NAIP5 [file cr2017148x4.pdf]

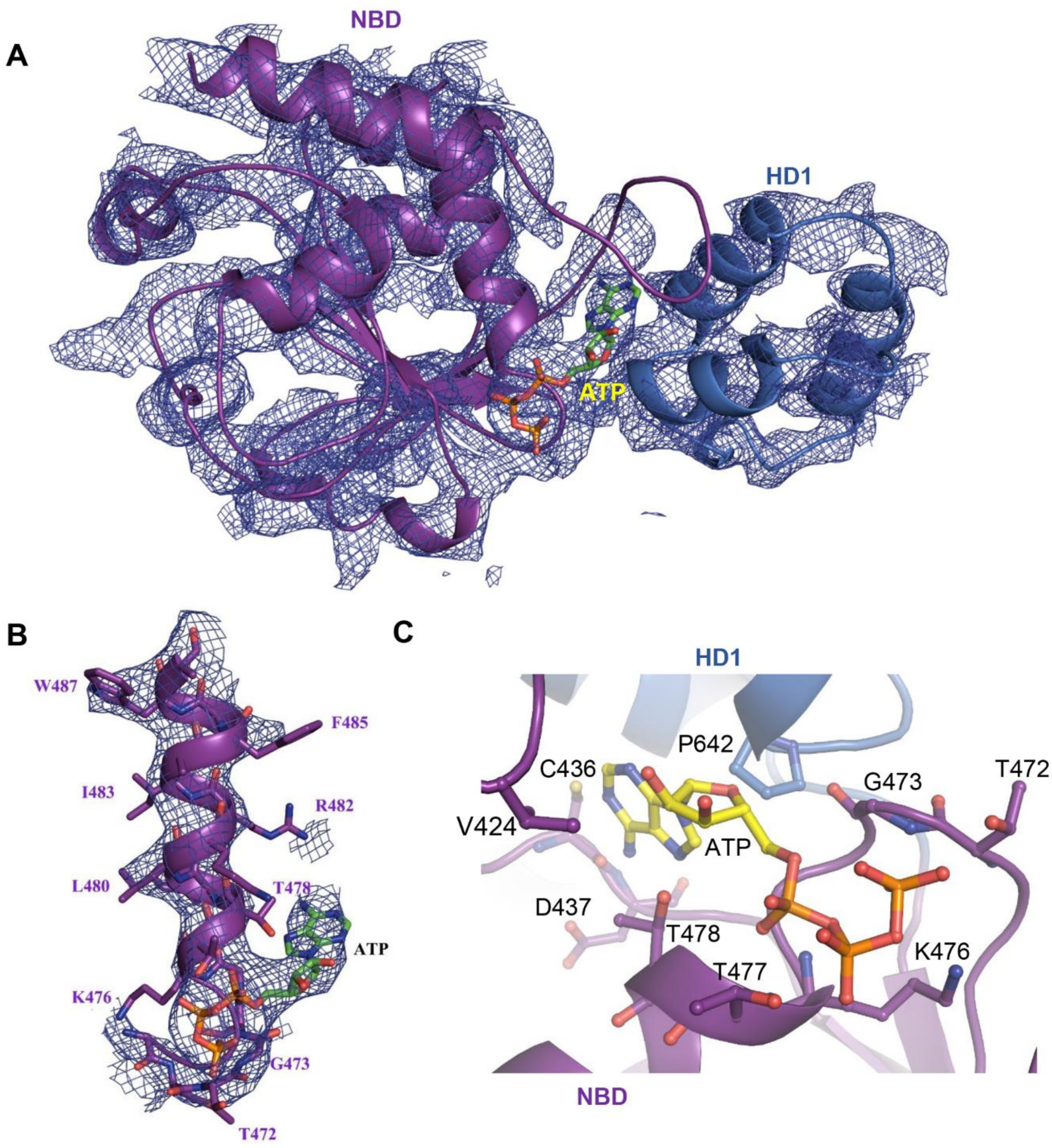
A
NBD
HD1
ATP
B
W487
F485
I483
R482
L480
T478
ATP
K476
G473
T472
C
HD1
P642
C436
G473
T472
V424
ATP
D437
T478
T477
K476
NBD

Supplementary information, Figure S4. Interaction of ATP with the FliC_D0$_L$-bound NAIP5

(A) The EM density around NBD and HD1 of NAIP5. ATP is shown in stick and labeled in yellow.

(B) The EM density around P-loop region of NAIP5.

(C) Interaction of ATP with its neighboring residues from NBD and HD1 of NAIP5.
