# Supplementary material for: Structural basis for specific flagellin recognition by the NLR protein NAIP5
Source: Cell Res. 2017 Nov 28;28(1):35–47. doi: 10.1038/cr.2017.148 (PMC5752844; doi:10.1038/cr.2017.148)
Supplement: Supplementary information, Figure S5 — Representative EM density maps for different structural domains from the FliC_D0L-NAIP5-NLRC4M complex [file cr2017148x5.pdf]

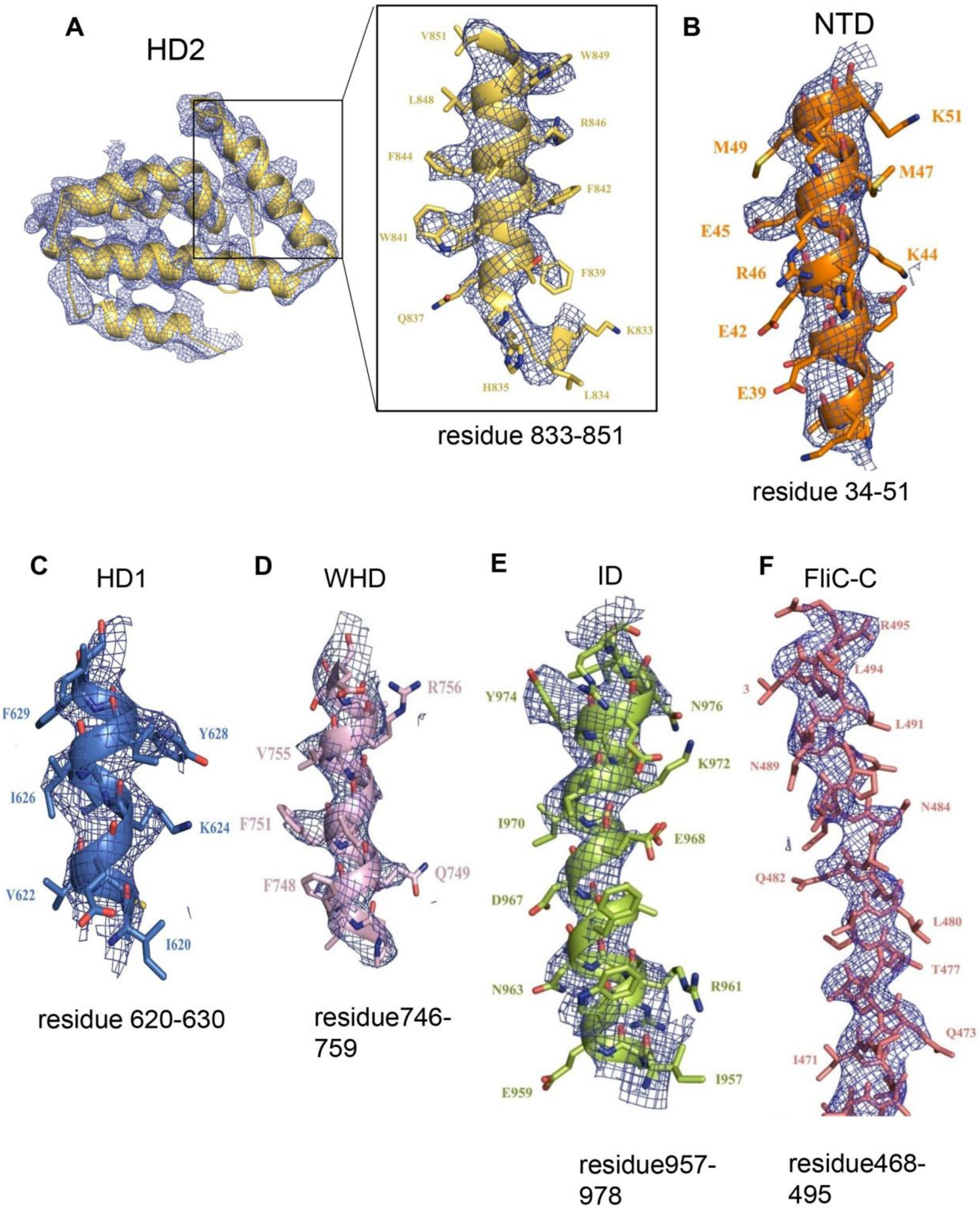

A
HD2
V851
W849
L848
R846
F844
F842
W841
F839
Q837
K833
H835
L834
residue 833-851
B
NTD
K51
M49
M47
E45
K44
R46
E42
E39
residue 34-51
C
HD1
F629
Y628
I626
K624
V622
I620
residue 620-630
D
WHD
R756
V755
F751
Q749
F748
residue746-759
E
ID
Y974
N976
K972
I970
E968
D967
N963
R961
E959
I957
residue957-978
F
FliC-C
R495
L494
L491
N489
N484
Q482
L480
T477
Q473
I471
residue468-495

Supplementary information, Figure S5. Representative EM density maps for different structural domains from the FliC_D0$_L$ -NAIP5-NLRC4^M complex

(A) Electron density around HD2 (left) and one α-helix of this domain (right).

(B) The EM density map around the second α-helix of NTD.

(C) The EM map density around one α-helix of HD1.

(D) The EM map density around one α-helix of WHD.

(E) The EM density around the C-terminal α-helix of ID.

(F) The EM density around the C-terminal α-helix of FliC_D0$_L$.
