# Supplementary material for: Structural basis for specific flagellin recognition by the NLR protein NAIP5
Source: Cell Res. 2017 Nov 28;28(1):35–47. doi: 10.1038/cr.2017.148 (PMC5752844; doi:10.1038/cr.2017.148)
Supplement: Supplementary information, Figure S6 — Structural superposition of NAIP5-NLRC4 with NLRC4 inflammasome [file cr2017148x6.pdf]

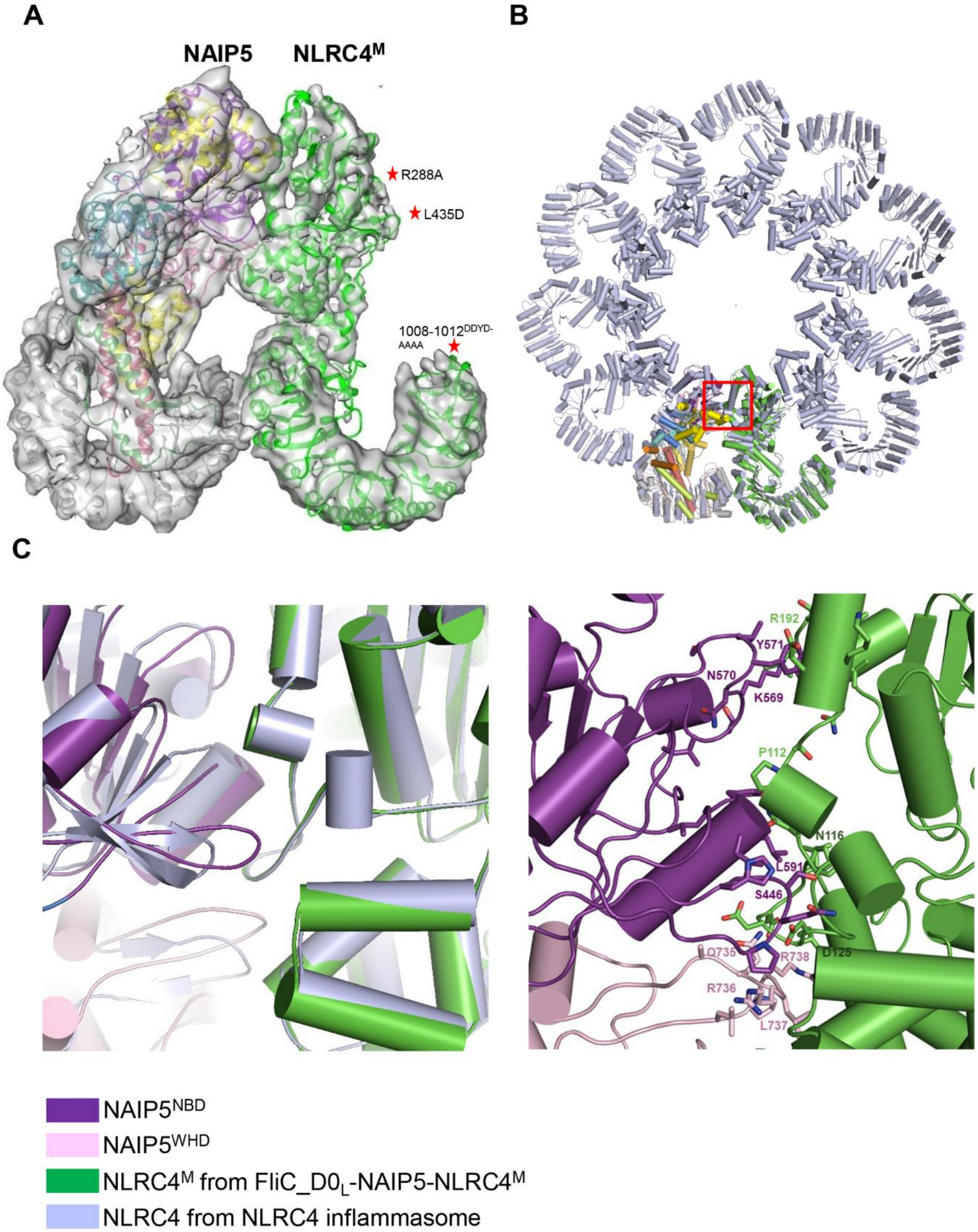

A
NAIP5
NLRC4M
R288A
L435D
1008-1012DDYD-AAAA
B
C
R192
Y571
N570
K569
P112
N116
L591
S446
Q735
R738
D125
R736
L737
NAIP5NBD
NAIP5WHD
NLRC4M from FliC_D0L-NAIP5-NLRC4M
NLRC4 from NLRC4 inflammasome

Supplementary information, Figure S6. Structural superposition of NAIP5-NLRC4 with NLRC4 inflammasome

(A) Docking of NAIP5 (left) and active NLRC4 (right) into the final electron density map of class5 particles (see Figure S2). Red stars indicate mutations in NLRC4^{M}. Those mutations introduced to catalytic surface prevent the latter NLRC4 being activated and result in a heterotrimer of FliC_D0$_{L}$ - NAIP5-NLRC4^{M} complex.

(B) Structural alignment of NAIP5-NLRC4^{M} from (A) with NLRC4 inflammasome. NLRC4^{M} from NAIP5-NLRC4^{M} was used to align an NLRC4 protomer from NLRC4 inflammasome (11mer). The region highlighted within the red frame is shown in (C).

(C) Left panel: The close-up view of structural comparison between the interface of NAIP5-NLRC4^{M} and that of NLRC4-NLRC4 around the NBD and WHD. NBD and WHD of NAIP5 (NAIP5^{NBD} and NAIP5^{WHD}) are shown in purple and pink, respectively, NLRC4^{M} from NAIP5-NLRC4^{M} in green and the two NLRC4 protomers from NLRC4 inflammasome in slate. Right panel: some detailed information of the interface between NAIP5 and NLRC4^{M}.
