# Supplementary material for: Structural basis for specific flagellin recognition by the NLR protein NAIP5
Source: Cell Res. 2017 Nov 28;28(1):35–47. doi: 10.1038/cr.2017.148 (PMC5752844; doi:10.1038/cr.2017.148)
Supplement: Supplementary information, Figure S7 — BIR3 is likely involved in closure of the wheel-like structure of NAIP5-NLRC4 inflammasome [file cr2017148x7.pdf]

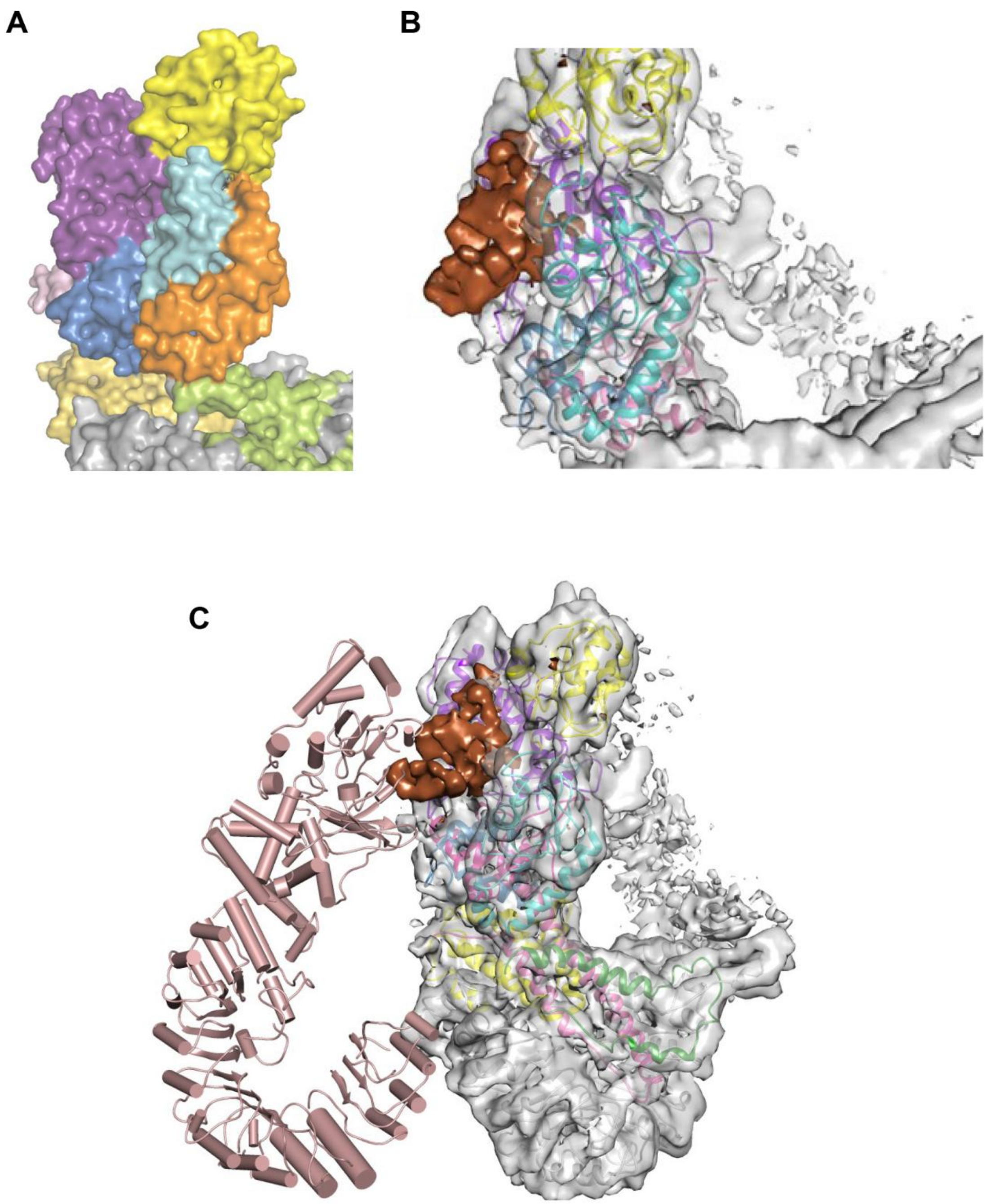
A
B
C

Supplementary information, Figure S7. BIR3 is likely involved in closure of the wheel-like structure of NAIP5-NLRC4 inflammasome

(A) Transparent surface representations of the large surface groove made by BIR1, BIR2, NBD and HD1. Color codes for domains are indicated

(B) The electron density map (transparent) around BIR1, BIR2, NBD and HD1 shown in cartoon. The density which likely corresponds to BIR3 is colored in brown.

(C) Structural superposition of a lateral NLRC4 dimer (cartoon) with NAIP5. The orientation of NAIP5 is the same as shown in (A).
