# Supplementary material for: Structural basis for specific flagellin recognition by the NLR protein NAIP5
Source: Cell Res. 2017 Nov 28;28(1):35–47. doi: 10.1038/cr.2017.148 (PMC5752844; doi:10.1038/cr.2017.148)
Supplement: Supplementary information, Figure S8 — Structural comparison of BIR1, BIR2 of NAIP5 and BIR3 of XIAP. [file cr2017148x8.pdf]

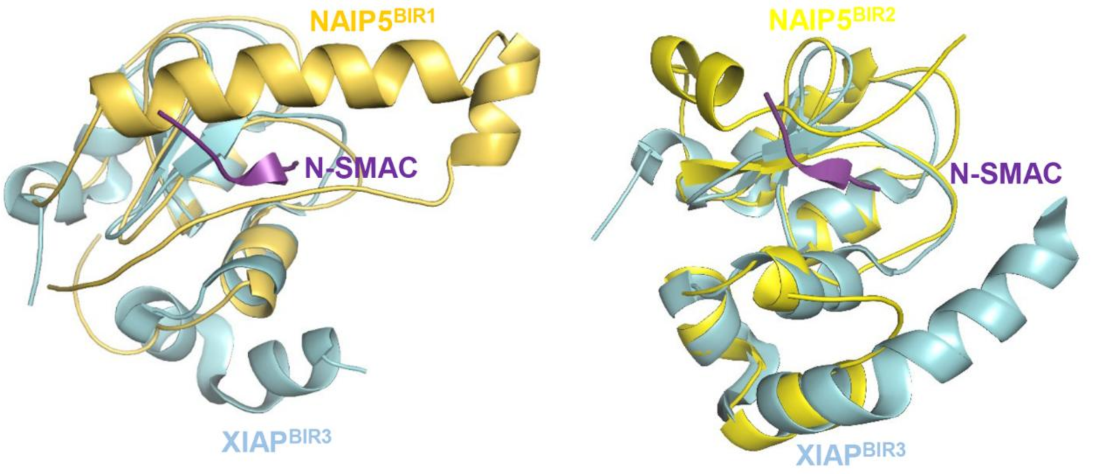
NAIP5BIR1
N-SMAC
XIAPBIR3
NAIP5BIR2
N-SMAC
XIAPBIR3

Supplementary information, Figure S8. Structural comparison of BIR1, BIR2 of NAIP5 and BIR3 of XIAP.

Structural superposition of $NAIP5^{BIR1}$ (left, light orange) and $NAIP5^{BIR2}$ (right, yellow) with $XIAP^{BIR3}$ (cyan). The $XIAP^{BIR3}$-bound peptide is from the N-terminal side of SMAC (N-SMAC) and shown in purple.
