# Supplementary material for: Structural basis for specific flagellin recognition by the NLR protein NAIP5
Source: Cell Res. 2017 Nov 28;28(1):35–47. doi: 10.1038/cr.2017.148 (PMC5752844; doi:10.1038/cr.2017.148)
Supplement: Supplementary information, Figure S9 — Structure-based sequence alignment of mouse NAIPs. [file cr2017148x9.pdf]

(A)

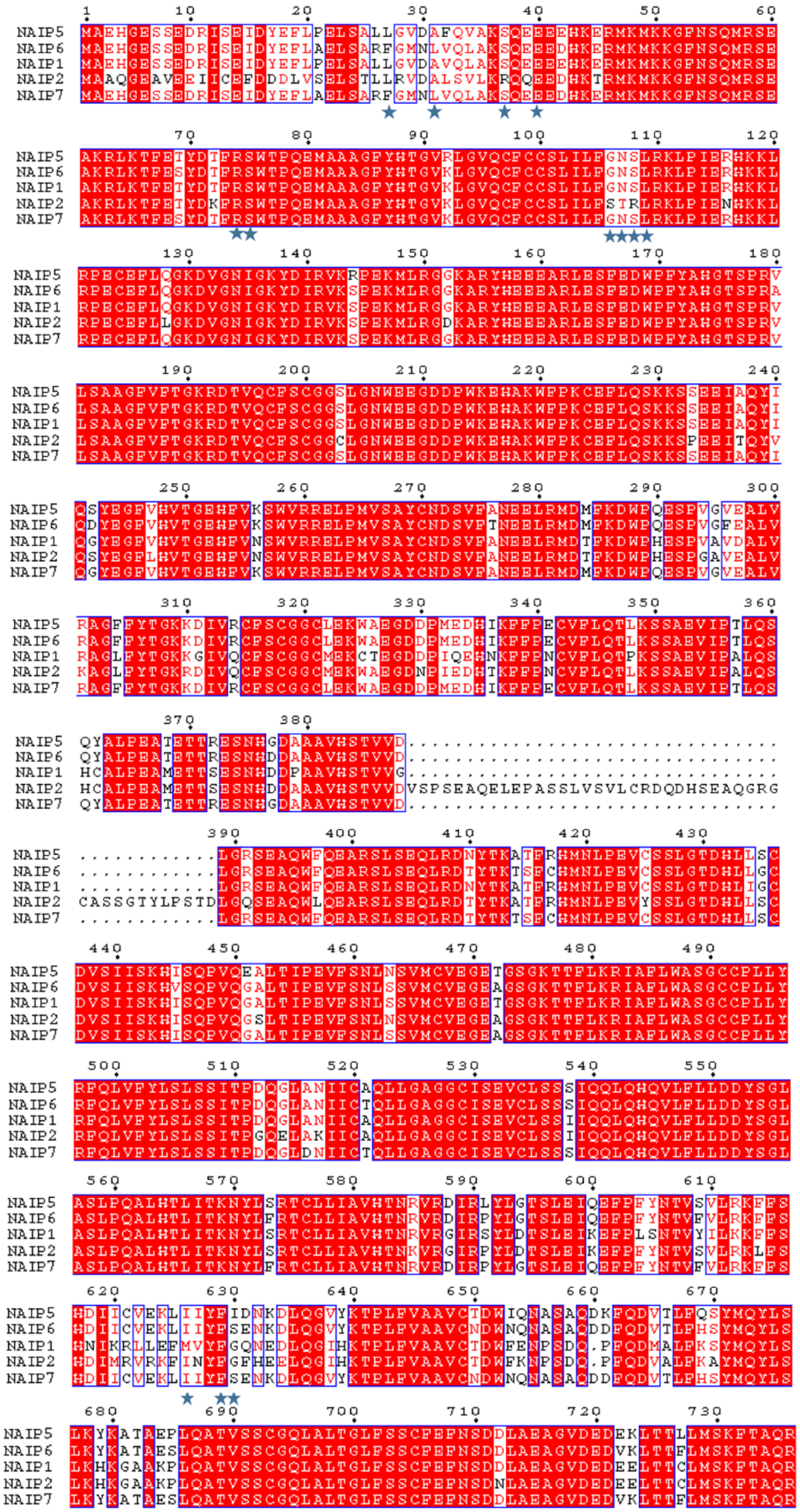

(B)

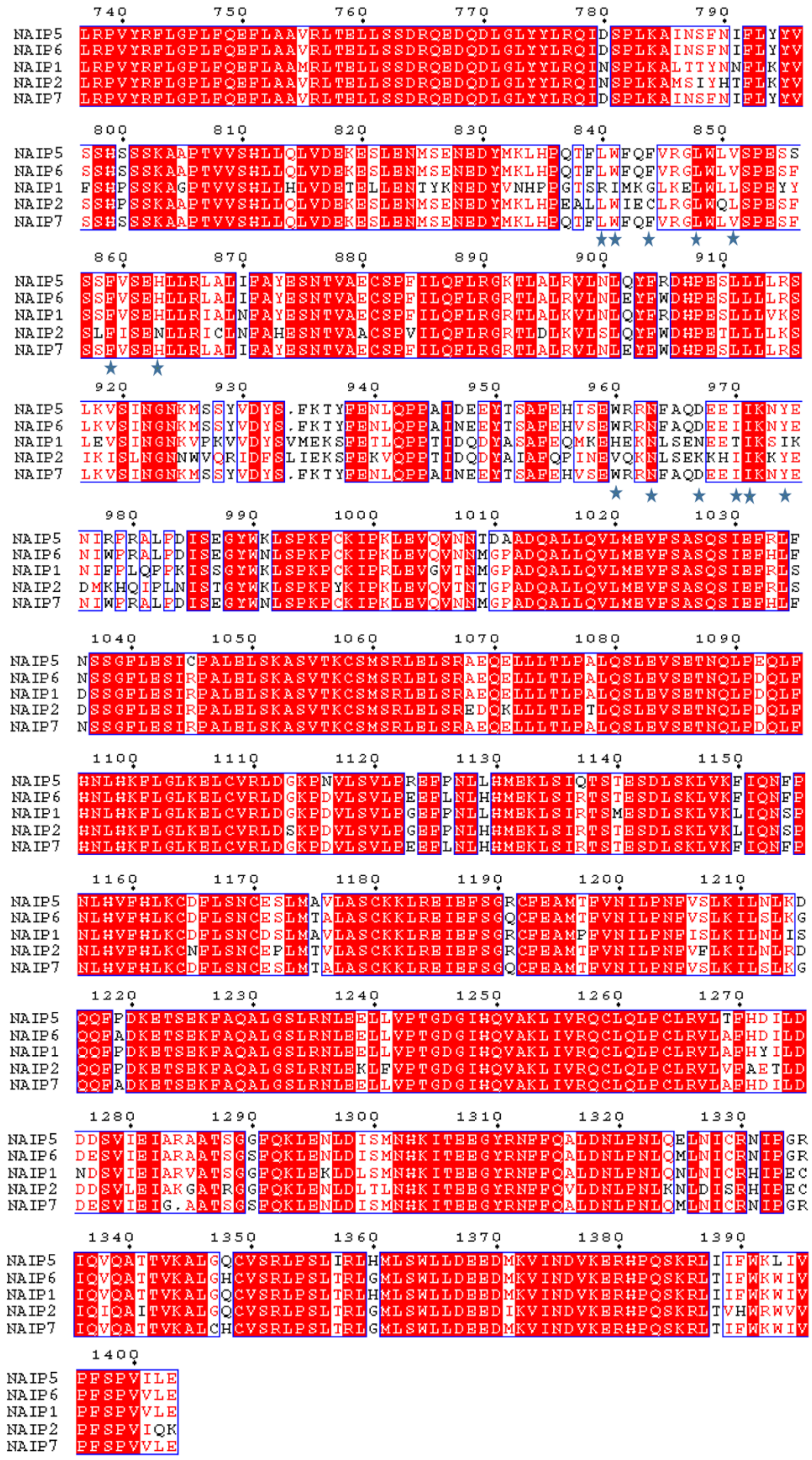

Supplementary information, Figure S9. Structure-based sequence alignment of mouse NAIPs.

Conserved and similar residues are highlighted in white and red, respectively. The stars indicate FliC_D0$_L$-interacting residues.
