# Supplementary material for: Structural basis for specific flagellin recognition by the NLR protein NAIP5
Source: Cell Res. 2017 Nov 28;28(1):35–47. doi: 10.1038/cr.2017.148 (PMC5752844; doi:10.1038/cr.2017.148)
Supplement: Supplementary information, Figure S10 — Structural comparison between the active NAIP5 and a modeled inactive NAIP5 [file cr2017148x10.pdf]

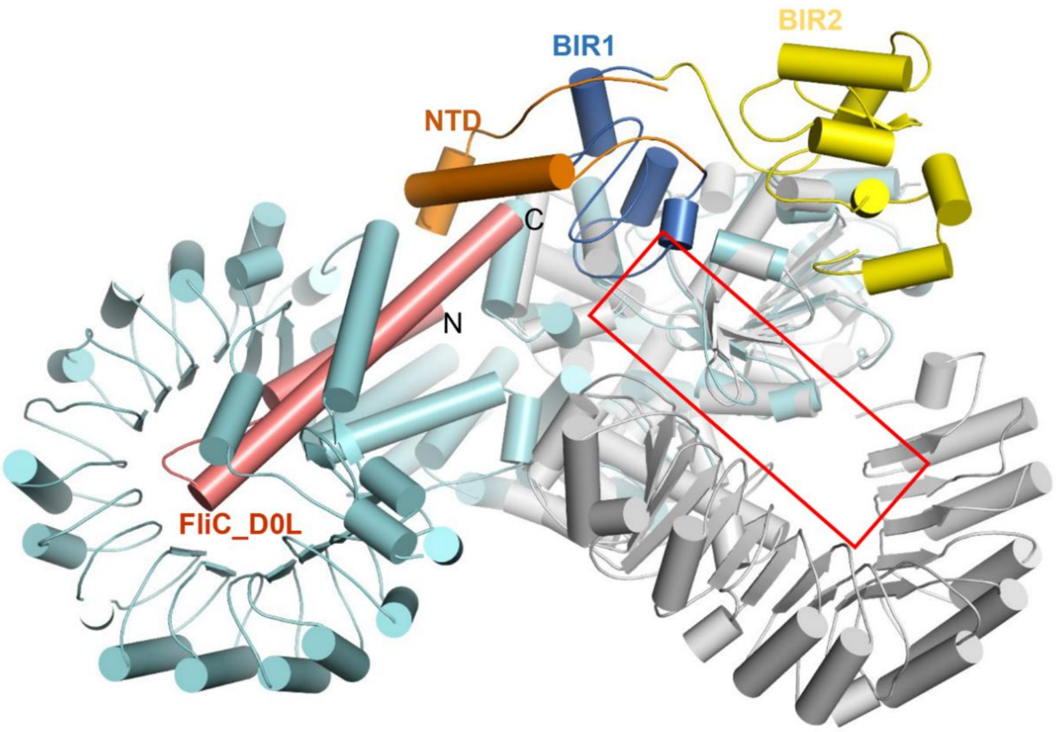
BIR2
BIR1
NTD
C
N
FliC_D0L

Supplementary information, Figure S10. Structural comparison between the active NAIP5 and a modeled inactive NAIP5

The structure of inactive NAIP5 (gray) was modeled using the program of MODELLER [53] with the inactive NLRC4 (PDB code: 4KXF). The NOD module (containing NBD and HD1) of the FliC_D0$_L$-bound NAIP5 was used as the template to align with the modeled structure of inactive NAIP5. The relative positioning of LRR and NBD in inactive NAIP5 could act to block the surface (highlighted within the red rectangle frame) where the C-terminal side of FliC-C (the longer helix) binds. For clarity, all the structural domains of NAIP5 are shown in cyan except that NTD, BIR1 and BIR2 are shown in orange, blue and yellow, respectively. “N” and “C” represent N- and C-terminus of FliC_D0$_L$, respectively.

Reference:

53. A. Fiser, A. Sali, Modeller: generation and refinement of homology-based protein structure models. *Methods Enzymol* **374**, 461-491 (2003)10.1016/S0076-6879(03)74020-8).
