# Supplementary material for: Structural basis for specific flagellin recognition by the NLR protein NAIP5
Source: Cell Res. 2017 Nov 28;28(1):35–47. doi: 10.1038/cr.2017.148 (PMC5752844; doi:10.1038/cr.2017.148)
Supplement: Supplementary information, Table S1 — Data collection and model statistics. [file cr2017148x11.pdf]

Supplementary information, Table S1. Data collection and model statistics.

**Model**	**NAIP5-FliC_D0$_L$**	
**Data collection**		
Electron microscope	FEI Titan Krios	
Voltage (kV)	300	
Electron Detector	Gatan K2 camera	
Pixel size (Å)	1.30654	
Electron dose(e-/ Å)	50 (32 frames)	
Defocus range(μm)	1.5~3.0	
**3D Reconstruction**		
Software	Relion-1.4	
Class	class5	class6
Particles for final refinement	245,315	381,293
Symmetry	C1	
Resolution (motioncor1, Å)	6.53	7.90
Resolution (motioncor2, Å)	4.93	4.58
Resolution (motioncor2+mask, Å)	4.42	4.51
Resolution (motioncor2+mask,,merge Å)	4.28	
Map sharpening B-factor (Å2)	-250	
**Model building**		
Software	COOT	
**Refinement**		
Software	PHENIX, VMD	
Resolution (Å)	4.28	
CC	0.740	
**Model composition**		
Protein residues	1313	
ATP	1	
**Validation**		

**R.m.s deviations**	
Bonds length (Å)	0.008
Bonds Angle (°)	1.175
**Ramachandran plot statistics (%)**	
Preferred (%)	89.98%
Allowed (%)	9.56%
Outlier (%)	0.46%
**Completeness of model**	
Amino acid residues defined by electron density	Chain A: S^{404}-E^{1403} Chain B: A^{10}-V^{247} Chain C: A^{421}-R^{495}
Except for amino acid residues[1]	H^{248}-L^{403}
Except for side chains[2]	Chain C: A^{421}-A^{423}

[1] Residues not included in the model

[2] Occupancy set to 0.00 in the model
